# Evaluation of polycyclic aromatic hydrocarbon contents in marine products in South Korea and risk assessment using the total diet study

**DOI:** 10.1007/s10068-023-01491-y

**Published:** 2024-01-09

**Authors:** Yujin Paik, Hoe-Sung Kim, Yong-Sung Joo, Jin Won Lee, Kwang-Won Lee

**Affiliations:** 1 Department of Biotechnology, College of Life Sciences and Biotechnology, Korea University, Seoul, 02841 Republic of Korea; 2 Department of Statistics, College of Natural Science, Dongguk University, Seoul, 04620 Republic of Korea; 3 Department of Bio Medicinal plant, Suwon Women’s University, Suwon-si, Gyeonggi-do 16632 Republic of Korea; 4 Division of Food Bioscience & Technology, College of Life Science and Biotechnology, Korea University, 212 CJ Food Safety Hall, Anam-Dong, Sungbuk-Gu, Seoul, 02841 Republic of Korea

**Keywords:** Polycyclic aromatic hydrocarbon, Marine product, Total diet study, Risk assessment, Gas chromatography-mass spectrometry

## Abstract

This study investigated levels of eight polycyclic aromatic hydrocarbons (PAH8) compounds in both raw and processed marine products in South Korea. Katsuobushi exhibited the highest concentration of benzo[a]pyrene, at 14.22 µg/kg, exceeding the European Commission's regulation level of 5.0 µg/kg. The total PAH8 concentration in katsuobushi was 220.5 µg/kg. Among the product categories, shellfish had the highest detection rate (70%), followed by fish (19%) and crustacea (8%), with chrysene being the most prominent PAH8 congener in all marine products. Grilled fish predominantly contained pyrogenic PAHs from combustion byproducts, while shellfish primarily contained petrogenic ones from the aquatic environment. Grilling, smoking, and drying processes significantly contributed to the formation of PAH8 in these food products. Based on the results of a risk assessment using a margin of exposure approach through a total diet study, exposure to PAH8 from marine products is considered to pose low concern to the South Korean population.

**Supplementary Information:**

The online version contains supplementary material available at 10.1007/s10068-023-01491-y.

## Introduction

Polycyclic aromatic hydrocarbons (PAHs) can come from three sources: natural (biogenic), combustion (pyrogenic), and petroleum (petrogenic) (Abbas et al., 2018). Biogenic PAHs stem from living organisms, while pyrogenic PAHs result from the high-temperature processing of organic matter. Petrogenic PAHs enter the environment through natural processes like gas leaks and fossil fuel seepage (Ofosu et al, 2022).

Many PAHs are mutagenic and genotoxic, capable of causing DNA adduct formation in both laboratory and living organisms (Zaidi, et al., 2021). In addition to cancer, PAHs can lead to other adverse effects, including neurobehavioral changes in developing animals, decreased ovarian follicles and ovary weight, and altered thymus weight and serum immunoglobulin in rats (Kitts et al., 2012; Kroese et al., 2002).

The European Food Safety Authority (EFSA) indicated that utilizing the total PAH level of benzo[a]anthracene (B[a]A), chrysene (Chry), benzo[a]pyrene (B[a]P), and benzo[b]fluoranthene (B[b]F) represented for PAH4, or including B[a]A, chrysene, B[a]P, B[b]F, benzo[k]fluoranthene (B[k]F), indeno[1,2,3-cd]pyrene (I[c,d]P), dibenz[a,h]anthracene (D[a,h]A), and benzo[g,h,i]perylene (B[g,h,i]P) represented for PAH8 is more appropriate than using solely B[a]P level (EC, 2015). The primary human exposure factor is known to be dietary intake, and the risk of exposure to PAHs in aquatic products has increased due to higher seafood consumption and pollution of the aquatic environment by human activities (Alomirah et al., 2011; Llobet et al., 2006). Other studies also found that seafood is becoming the leading source of dietary PAH exposure (EFSA, 2008; Habibullah-Al-Mamun et al., 2019; Veyrand et al., 2013).

As a result, we employed a total diet study (TDS) approach to assess the levels of PAHs in marine products and investigate variations in PAH contents based on the preparation methods of these marine food items. TDS is one of the most effective and efficient methods for estimating dietary exposure and assessing health risk at the consumer level, considering the purchasing and eating habits of various populations (Ingenbleek et al., 2017). There are limited studies that monitor the levels of benzopyrene, the total of PAH4 (∑PAH4), and the total of PAH8 (∑PAH8) generated during the processing of marine products. In the case of barbecued salmon fillet and grilled anchovies, it was observed that they exhibited elevated levels of benzo[a]pyrene, the total of PAH4 (∑PAH4), and the total of PAH8 (∑PAH8), with concentrations of 0.52 µg/kg, 2.41 µg/kg, and 2.88 µg/kg for the former (Oz, 2020), and 0.73 µg/kg, 3.3 µg/kg, and 5.13 µg/kg for the latter (Sahin et al., 2020).

Because the toxicity of each PAH differs, the toxicity is assessed using a toxic equivalency factor (TEF) using benzo[a]pyrene as a reference molecule. EFSA adopted a margin of exposure (MOE) approach to estimate health risk to PAHs, based on benchmark dose lower-bound confidence limit 10% (BMDL_10_) (EFSA, 2008). However, EFSA has concluded the toxic equivalent quantity (TEQ) approach is only suitable for compounds with the same toxicological effect, such as dioxins. Given that several PAHs have carcinogenic properties and produce tumors through different mechanisms, the TEQ approach may not be appropriate for PAHs (EFSA, 2008). Using toxicological values, rather than the TEQ, may be necessary for assessing the risks associated with PAHs.

The primary objectives of this research are to: (1) validate methods for extracting PAHs to evaluate the PAH8 concentration, (2) identify the main contributors of PAH8 exposure in humans in terms of each PAH compound and the origin of PAHs associated with processing methods, (3) determine the types of marine products generating most PAH8, (4) assess the health risks associated with PAH8 from the consumption of marine products in South Korea, and (5) to compared the risks estimated using toxicological values and traditional TEF values. This study focused on examining the PAH8 levels in marine products in a table-ready form within the framework of a TDS.

## Materials and methods

### Sample selection and preparation

Marine product samples were selected based on the results of the 6th Korean National Health and Nutrition Examination Survey (KNHANES-VI) conducted by Korea Disease Control and Prevention Agency (KDCA) from 2013 to 2015 (MOHW, 2013). The raw data were obtained from the KNHANES website (https://knhanes.kdca.go.kr/knhanes/main.do).

Supplementary Table 1 contains a list of 109 products with high consumption rates (covering more than 95% of total accumulated consumption), high frequency levels (covering more than 1% of the consumption rate), and fat contribution (containing the cumulative rate of fat intake up to 95%). The food samples were gathered from 21 supermarkets in ten large cities with a population of over one million, including Seoul, Incheon, Suwon, Gwangju, Daejeon, Cheongju, Daegu, Busan, Ulsan, and Changwon in South Korea.

An identical quantity of food samples were gathered to create each composite sample, and they were prepared in a table-ready form according to the sample preparation methods guidebook for TDS provided by the Ministry of Food and Drug Safety (MFDS, 2019). The cooked samples (n = 287) are divided into eight groups: fish (139), shellfish (46), cephalopoda (25), crustacea (37), sea algae (30), echinodermata (5), tunicata (3), and cnidaria (2). A total of 287 subsamples were homogenized and kept in a polyethylene bottle at − 20 °C for PAH analysis.

### Chemicals and materials

HPLC grade ethyl alcohol, methyl alcohol, n-hexane, and dichloromethane (DCM) were purchased from Burdick & Jackson (Muskegon, MI, USA). Younglin Instrument's AQUAMAX-Basic 363 water purification system (Dongan, Anyang, Republic of Korea) was used to produce distilled water. EPA 525 PAH Mix A, which contains 500 µg/mL of naphthalene (99% purity), acenaphthene (99% purity), fluoranthene (98% purity), acenaphthylene (99% purity), fluorene, phenanthrene, anthracene, pyrene, benz[a]anthracene, chrysene, benzo[b]fluoranthene (98% purity), benzo[k]fluoranthene, benzo[a]pyrene (96% purity), indeno[1,2,3-cd]pyrene, dibenz[a,h]anthracene, and benzo[g,h,i]perylene in dichloromethane was purchased from Sigma Aldrich (St. Louis, MO, USA). Benzo[a]pyrene-d_12_ (98% purity), benzo[a]pyrene-d12 (98% purity), benzo[b]fluoranthene-d12 (98% purity), and chrysene-d_12_ (98% purity) were purchased from Sigma Aldrich for internal standards. Potassium hydroxide was purchased from Showa Denko (Tokyo, Japan), and anhydrous sodium sulfate was from Yakuri Pure Chemicals (Kyoto, Japan). Filter paper was purchased from Whatman (Kent, UK) and Bond Elut SI for solid phase extraction (SPE) was obtained from Agilent Technologies (Santa Clara, CA, USA).

### Extraction of PAHs

Extraction steps are essential for determining PAHs. Depending on the properties of the sample matrices, two distinct extraction procedures were employed. These methods were adapted in accordance with the Korea Food Code and previous studies (Kim et al., 2021; MFDS, 2019).

For solid food matrices such as salmon and oyster, alkali digestion was applied to extract PAHs from the products. In each flask, 10 g (wet weight) of samples or 1–2 g (dry weight) of sample were placed. Then, 100 mL of 1 M KOH solution in ethanol was added, along with 1 mL of ^13^C-labeled internal standard solution (100 µg/kg of each benzo[a]pyrene-d_12_ and chrysene-d_12_). The flask was connected to a reflux condenser and placed into a water bath, WB-22, (Daihan Scientific, Gangwon, Republic of Korea) at 80℃ for 3 h. After saponification, the flask was rapidly cooled down using cold water, and the reflux condensers were rinsed with n-hexane. The extract in the flask was transferred into a separatory funnel through a filter paper. The flask was washed with 50 mL of n-hexane: ethanol (1:1, v/v) solution, and the washed solution was added into the funnel. The funnel was thoroughly shaken using a funnel shaker (Changshin Science, Seoul, Republic of Korea) with 300 rpm for 10 min after adding 50 mL of distilled water. After shaking, the separated organic phase fraction from the organic solvent was collected in each Erlenmeyer flask. Then, 50 mL of n-hexane was added to distilled water in the separatory funnel and shaken to separate two immiscible liquid phases. This procedure was repeated twice. All organic solvent phases were collected in another separatory funnel, and distilled water was added to remove water-soluble compounds. The water layer was eliminated after vigorously shaking the funnel. The obtained extract was filtered through 10 g of anhydrous Na_2_SO_4_ (Yakuri Pure Chemicals, Kyoto, Japan) to remove any remaining water. Next, the extracts were concentrated using a rotary evaporator (EYELA, Tokyo, Japan) until the final volume was below 2 mL. The concentrate was applied to activate SPE (Bond Elut SI) cartridges (Agilent technologies) and eluted with n-hexane and DCM. The eluant was concentrated under N_2_ gas at 40 °C, and the residues were re-dissolved in 1 mL of DCM. The solution was filtered through 0.45 µm of PTFE membrane syringe filter for gas chromatography-mass spectrometry (GC–MS) analysis.

For liquid food matrices, such as fish sauce or seafood stock, an ultrasound-assisted extraction method was used to quantify PAHs. In each flask, 10 g of samples were placed, and 50 mL of n-hexane spiked with 1 mL of internal standard solution was added. The mixture was then subjected to 20 min of ultrasonication. Subsequently, 35 mL of n-hexane was added, and ultrasound-assisted extraction was carried out for another 20 min. The following extraction steps for liquid food samples were conducted in the same manner as previously described methods for solid food samples.

### GC–MS analysis

PAH levels were determined using gas chromatography-mass spectrometry (GC–MS, 7890B/5977B, Agilent Technologies) with a HP-5MS Ultra Inert column (30 m × 0.25 mm × 0.25 μm) (Agilent Technologies). Helium was used as a carrier gas with a flow rate 1.0 mL/min. The GC oven temperature was programmed as follows: initially set at 80 °C and held for 1 min, then heated at a rate of 20 °C/min up to 220 °C and held for 10 min, followed by an increase to 280 °C at a rate of 2 °C/min and maintained for 10 min. Mass spectra were generated using an electron ionization ion source at 70 eV in scan mode to determine a quantitative ion and two qualitative ions (Table 1). PAH peaks were identified using the ion in the selected ion monitoring mode.

**Table 1:** Summary of eight polycyclic aromatic hydrocarbons (PAH8) and validation results of two different methods (alkali digestion and ultrasound-assisted extraction) with linearity, limit of detection (LOD), limit of quantification (LOQ), and expanded uncertainty

Compounds	Abbreviation	Quantitative ion (m/z)	Qualita-tive ion (m/z)	TEF^a^	Alkali digestion extraction method				Ultrasound assisted extraction method			
					R^2^	LOD^b^ (µg/kg)	LOQ^c^ (µg/kg)	Expanded uncertainty^d^ (%)	R^2^	LOD (µg/kg)	LOQ (µg/kg)	Expanded uncertainty (%)
Benz[a]anthracene	B[a]A	228	226, 229	0.1	0.9998	0.096	0.292	4.022	0.9991	0.079	0.241	4.463
Chrysene	Chry	228	226, 229	0.01	0.9996	0.082	0.248	6.145	0.9992	0.074	0.225	6.196
Benzo[b]fluoranthene	B[b]F	252	250, 253	0.1	0.9997	0.091	0.277	5.649	0.9999	0.083	0.252	5.603
Benzo[k]fluoranthene	B[k]F	252	250, 253	0.1	0.9999	0.120	0.365	6.485	0.9995	0.105	0.317	7.061
Benzo[a]pyrene	B[a]P	252	250, 253	1	0.9994	0.070	0.212	4.511	0.9998	0.115	0.348	4.495
Indeno[1,2,3-cd]pyrene	I[c,d]P	276	274, 277	0.1	0.9999	0.129	0.391	4.784	0.9997	0.108	0.327	4.995
Dibenz[a,h]anthracene	D[a,h]A	278	276, 279	5	0.9999	0.175	0.531	4.447	0.9998	0.097	0.294	4.615
Benzo[g,h,i]perylene	B[g,h,i]P	276	274, 277	0.01	0.9983	0.105	0.318	8.608	0.9984	0.104	0.315	8.529

^a^Toxic equivalency factors (TEFs)

^b^Method limit of detection, 3.3σ/s (σ = standard deviation of response; s = slope of regression equation)

^c^Method limit of quantification, 10σ/s

^d^Expanded uncertainties in percentage at 10 μg/kg (confidence level about 95%, *k* = 2). If nominal concentration level is < 100 μg/kg, the typical expanded uncertainty range should be within 44% (Codex, 2011)

### Method validation

Salmon (solid-type) and fish sauce (liquid-type) were utilized as representative samples for each alkali digestion method and ultrasound-assisted extraction method to validate the efficacy of these two distinct PAH extraction procedures. Linearity, limit of detection (LOD), limit of quantification (LOQ), accuracy, precision, and measurement uncertainty were assessed to validate both PAH extraction methods. Additionally, proficiency testing using Food Analysis Performance Assessment Scheme (FAPAS) was also conducted.

Calibration curves for eight targets of PAHs (PAH8) were obtained through five replicate experiments with 6 data points spanning the range of 0 to 20 µg/kg range, each spiked with the 100 µg/kg of ^13^C-labeled internal standard solution. Linearity, LOD, and LOQ were determined by plotting the ratios of analyte compound peak area to their corresponding internal standards against nominal concentrations (0, 0.5, 1, 2, 5, 10, and 20 µg/kg). The linearities of each calibration curve were assessed as a coefficient of determination (R^2^). The LOD and LOQ were calculated using the following formula: LOD = 3.3σ/S and LOQ = 10σ/S, where σ represents the standard deviation of the response, and S is the slope of the calibration curve. Accuracy and precision were assessed at three different nominal concentration levels (5, 10, and 20 µg/kg), with intraday accuracy and precision determined from five replicates and interday accuracy and precision validated in triplicate across three days.

To ensure the reliability of our analysis data, the measurement uncertainty was calculated, following the EURACHEM/CITAC Guide (EURACHEM/CITAC, 2012). This involved evaluating the standard uncertainty of each factor affecting the measurement value, including balance, pipette, volume of mass flask, external standard solution, internal standard solution, calibration curve, matrix effects, and GC–MS. For determining the measurement uncertainty, 10 µg/kg of external standard solution and 100 µg/kg of internal standard solution were used. After assessing each standard uncertainty, they were integrated to obtain a combined standard uncertainty (*u’*). An expanded measurement uncertainty (U’) was calculated by multiplying *u’* by a coverage factor (*k* = 2), representing a confidence level of approximately 95%.

Our laboratory’s performance for PAH quantitation was evaluated trough FAPAS proficiency testing, with results falling within the range of |z|< 2.

### Exposure assessment and risk characterization

Various statistical treatments were employed to handle findings below the LOD depending on the detection rate. In accordance with JOINT GUIDANCE (FAO, 2011), it is typically recommended for risk assessment to use both a lower bound (LB) and an upper bound (UB). Results falling below LOD are substituted for zero at the LB and replaced by LOD at the UB (EFSA, 2008; WHO/IPCS, 2009).

To calculate total PAH8 concentration (TC_PAH8_ or ∑PAH8), the concentrations of individual congener were combined by using Eq. (1). Meanwhile, to determine the total B[a]P toxic equivalent quantity (TEQ_B[a]p_) of PAH8, the concentrations of each PAH compound were multiplied by their toxic equivalency factors (TEFs) and then summed using Eq. (2). The TEFs used in this study are based on those reported by Nisbet and LaGoy (1992), which are presented in Table 1.

1 $${\text{TCPAH8i}} = \mathop \sum \limits_{{{\text{i}} = 1}}^{{\text{n}}} {\text{Ci }} \left( \frac{ng}{g} \right)$$

2 $${\text{TEQB[a]Pi}} = \mathop \sum \limits_{{{\text{i}} = 1}}^{{\text{n}}} {\text{Ci }} \times {\text{TEFi}} \left( \frac{ng}{g} \right)$$

where, TC_PAH8_ is the total concentration of the ith individual congener of PAH8, TEQ_B[a]Pi_ is the total B[a]P toxic equivalent concentration of the ith individual congener of PAH8, Ci is the measured concentration for the i^th^ individual congener of PAH8, and TEF_i_ is the toxic equivalency factor of the ith individual congener.

The daily intakes of TC_PAH8_ and TEQ_B[a]P_ from marine food exposure were calculated using Eq. (3) and (4), respectively. These equations multiply the food intake rate (IR_i_) by the TC_PAH8_ or TEQ_B[a]P_ value and divide by the body weight (b.w.). The respective IR_i_ and BW of total population and consumption group were obtained from the KNHANES published by the KDCA.

3 $${\text{Dietary exposure }} = { }\mathop \sum \limits_{{{\text{i}} = 1}}^{{\text{n}}} \frac{{{\text{TCPAH8i}} \times {\text{IRi }}}}{{{\text{b}}.{\text{w}}}}{ }\left( {\frac{{{\text{ng}}}}{{{\text{kg b}}.{\text{w}}.{\text{day}}}}} \right)$$

4 $${\text{Dietary exposure }} = { }\mathop \sum \limits_{{{\text{i}} = 1}}^{{\text{n}}} \frac{{{\text{TEQB[a]Pi }} \times {\text{IRi }}}}{{{\text{b}}.{\text{w}}.}}{ }\left( {\frac{{{\text{ng}}}}{{{\text{kg b}}.{\text{w}}.{\text{ day}}}}} \right)$$

To determine the daily exposure to PAH8 through marine food, the MOE was recommended by a Scientific Committee (EFSA, 2005). The MOE was calculated based on the TEQ approach, where a Benchmark Dose Lower Limit (BMDL_10_) of PAH8 was divided by the daily dietary exposure expressed in total concentration of PAH8 (∑PAH8) or total B[a]P equivalent quantity (TEQ_B[a]P_)) using the following Eq. (5). The BMD10 and BMDL10 values for PAH8 in the experimental animal diet ranged from 0.87 to 1.93 mg/kg b.w. per day and 0.49 to 1.35 mg/kg b.w./day, respectively. The CONTAM Panel used the lowest BMDL10 value of 0.49 mg/kg b.w. per day to derive an MOE, which measures how safe a chemical is at a given exposure level (EFSA, 2008). Thus, The BMDL_10_ of 0.49 mg/kg BW/d was chosen as the reference point of PAH8 in this study.

5 $${\text{MOE}} = { }\frac{{{\text{BMDL10}}\left( {\frac{{{\text{ng}}}}{{{\text{kg b}}.{\text{w}}.{\text{ day}}}}} \right)}}{{{\text{Daily exposure}}\left( {\frac{{{\text{ng}}}}{{{\text{kg b}}.{\text{w}}.{\text{ day}}}}} \right)}}$$

An MOE value lower than 10,000 is considered as a possible concern for risk management while an MOE of 10,000 or higher is regarded to indicate a low concern (EFSA, 2005, 2008).

## Results and discussion

### Method validation and quality control

The linearities (R^2^) of calibration curves after alkali digestion and ultrasound-assisted extraction, respectively were above 0.9983 (Table 1) satisfying the Codex guideline requirement which specifies that R^2^ should be 0.99 or above (Codex, 1993). While the LOD of the former pretreatment for PAH8 congeners ranged from 0.070 to 0.175 µg/kg, that of the latter method ranged from 0.074 to 0.115 µg/kg. The measurement uncertainty ranged from 4.022 to 8.608% and 4.463 to 8.529% in alkali digestion and ultrasound-assisted extraction methods, respectively. This result met the Codex criteria, which state that the expanded uncertainty should be less than 44% when the nominal concentration was 100 µg/kg or less (Codex, 2011). Additionally, standard uncertainty of calibration curve was found to be the most influential factor in determining the measurement uncertainty of PAH8. As shown in Table 2, the accuracy ranged from 91.83 to 111.8% and the precision were 0.07 to 8.75%. Based on these validation results, both extraction procedures were shown to be highly effective in determining the content of PAH8 in marine products.

**Table 2:** Accuracy and precision of PAH8 using two different extraction methods

Compounds	Nominal concentration (µg/kg)	Alkali digestion method				Ultrasonication method			
		Intra-day^a^ (n = 5)		Inter-day^b^ (n = 3)		Intra-day (n = 5)		Inter-day (n = 3)	
		Accuracy	Precision	Accuracy	Precision	Accuracy	Precision	Accuracy	Precision
		(%)	(% RSD^c^)	(%)	(% RSD)	(%)	(% RSD)	(%)	(% RSD)
B[a]A	5	97.35–100.00	1.92–3.42	99.74	1.09	98.43–101.39	0.53–2.27	100.70	0.84
	10	98.80–101.02	1.07–3.41	99.79	0.62	99.54–103.56	1.87–2.17	100.45	0.80
	20	99.98–106.03	1.87–4.08	102.13	2.28	102.18–106.83	1.57–2.43	102.11	1.50
Chry	5	98.30–100.27	0.76–0.99	99.85	0.68	98.38–100.72	1.05–2.26	99.77	0.53
	10	96.75–99.73	1.72–3.13	99.52	0.81	98.29–100.83	0.82–2.21	99.93	0.26
	20	99.50–106.62	0.93–7.42	100.01	0.24	102.41–103.05	0.80–3.71	100.77	3.32
B[b]F	5	97.92–98.47	1.20–2.59	99.93	0.47	96.82–98.15	0.55–1.20	98.60	0.66
	10	97.23–97.76	2.66–4.86	99.58	0.42	97.78–98.06	0.60–0.76	98.65	0.10
	20	98.24–105.74	1.12–8.35	100.15	0.08	98.35–98.92	0.39–0.57	99.20	0.31
B[k]F	5	99.07–100.30	0.87–2.49	99.99	0.07	96.29–100.39	1.32–4.22	98.82	0.60
	10	98.49–99.64	2.32–3.22	100.24	0.13	89.93–96.94	2.83–6.72	97.81	2.43
	20	99.95–101.91	0.24–1.25	100.23	0.38	96.26–97.18	1.12–2.60	98.74	0.89
B[a]P	5	98.80–100.52	1.03–1.87	99.96	0.25	98.79–101.00	1.20–3.76	100.60	0.68
	10	98.31–100.29	1.31–3.92	100.48	0.91	96.86–98.77	0.75–2.95	99.60	0.64
	20	99.98–111.30	0.96–8.75	100.41	0.36	97.84–99.72	0.65–1.27	99.86	0.48
I[c,d]P	5	98.92–102.63	1.13–4.87	99.76	0.75	91.83–93.76	0.64–1.77	94.44	1.71
	10	98.25–101.87	1.94–4.27	100.27	0.38	94.07–96.76	2.29–2.85	98.19	1.47
	20	101.47–105.78	1.38–3.31	101.17	1.55	94.20–95.55	1.97–2.44	97.54	1.69
D[a,h]A	5	99.85–105.53	0.78–7.00	101.37	0.71	94.62–96.16	1.53–7.36	98.88	1.65
	10	100.32–107.50	0.51–5.29	100.92	0.63	98.53–104.07	1.98–3.86	99.82	0.89
	20	102.97–111.82	2.88–6.44	103.99	3.43	99.54–106.69	1.04–7.22	100.47	1.12
B[g,h,i]P	5	99.23–105.51	1.22–7.10	101.03	1.41	92.71–93.11	0.61–1.36	94.01	0.69
	10	104.06–109.97	2.77–4.49	102.13	1.93	95.60–96.21	1.15–2.50	97.70	1.10
	20	100.83–108.58	1.67–5.75	101.38	0.87	98.29–100.09	1.18–1.85	99.68	1.15

Ranged from mean of 5 determinations performed daily for 3 days

Mean of 3 determinations

Relative standard deviation: 100× standard deviation/mean

### Concentration of PAH8 in marine products

According to the results of the determination PAH8 in marine products prepared as ready-to-eat, the number of samples and the number of detected samples in the eight categories of marine samples were as follows: Fish (n = 26 out of 139; the number of detected samples.

out of total samples), Shellfish (n = 32 out of 46), Cephalopods (n = 11 out of 25), Crustacea (n = 3 out of 37), Sea algae (n = 13 out of 30), Echinodermata (n = 5 out of 5), Tunicata (n = 2 out of 3), and Cnidaria (n = 1 out 2) (Supplementary Table 2). PAH8 levels were found to be above the limits of over the LODs in 93 of 287 subsamples. Among the tested samples, katsuobushi (dried and smoked bonito) exhibited the highest B[a]P levels, followed by dried sea cucumber (Supplementary Table 2). Katsuobushi showed the concentrations of B[a]P (14.22 μg/kg), B[a]A (70.95 μg/kg), Chry (90.63 μg/kg), B[b]F (24.62 μg/kg), B[k]F (8.482 μg/kg), I[c,d]P (4.924 μg/kg), D[a,h]A (1.277 μg/kg), and B[g,h,i]P (5.367 μg/kg) (data not shown).

However, both katsuobushi (smoked and dried bonito) and dried sea cucumber were found to contain levels of B[a]P, a known carcinogen (EFSA, 2008), that exceeded both EC (2015) and MFDS (2019) regulations. The EC regulation for B[a]P in smoked fish is 5 µg/kg, and the MFDS regulation is 5 µg/kg for smoked fish and 10 µg/kg for smoked and dried fish. Katsuobushi contained 14.22 µg/kg of B[a]P, and dried sea cucumber contained 11.35 µg/kg of B[a]P. Tsutsumi et al. (2019) demonstrated that 21 µg/kg B[a]P in dried bonito flakes demonstrated 1.5 times higher than the concentration in the present study. PAHs can form in the smoke that is produced during cooking, and they can also be formed on the surface of the food itself (Alomirah et al., 2011). Another study by Kafouris et al. (2020) found that smoked fish contained significantly higher levels of PAHs compared to fresh fish. The findings of these studies suggest that open flame cooking methods can elevate the B[a]P content of the smoked and dried katsuobushi. The sea cucumber samples in this study are dried. Dried sea cucumber is the most popular form of sea cucumber, accounting for 80% of the market. To reduce processing time, several drying methods have been developed, including hot-air drying at temperatures of 60–100 °C and vacuum cooking at 95 °C (Fan et al., 2022). The thermal processing of dried sea cucumber samples in this study may have increased the levels of B[a]P in the samples. For non-processed products other than smoked fish, the B[a]P level in the grilled salted mackerel was substantially higher (8.5 µg/kg). Open flame cooking methods, like grilling, are known to generate elevated levels of levels of PAHs in food, as PAHs are a class of organic compounds formed during the incomplete combustion of carbon-based materials such as wood, charcoal, and fat (Sampaio et al., 2017). How PAHs form in food during open flame cooking is a complex process that depends on a number of factors, such as the type of fuel used, the cooking temperature, and the cooking time. For instance, a study by Alomirah et al. (2011) found that grilled meat contained significantly higher levels of PAHs than baked meat. Consumers should be aware of the potential health risks associated with the consumption of PAHs and should consider alternative cooking methods, such as smoking, baking or roasting, whenever possible.

However, the shellfish category had the highest detection rate (70%) above LOD, while the fish and crustacea categories had low detection rates (19% and 8%, respectively). The different detection rates between shellfish and crustaceans were explained by their differing feeding habits. Shelfish feeds on suspended nutritional components in water, whereas crustaceans are scavengers (Veyrand et al., 2013). PAHs were found in all mussel subsamples, and the toxicants are thought to have come from contaminated sea water (EFSA, 2008). Although shellfish were frequently tested for contamination, none of the subsamples exceeded the regulatory limits set by EC and MFDS. According to the regulations from MFDS (2019), the maximum permissible levels of B[a]P in bivalvia and cephalopoda are 10.0 µg/kg and 5.0 µg/kg, respectively. Overall, there was a variation in PAH levels depending on cooking methods, with the smoke produced during the heating process appearing to be the primary cause of excessive PAH levels.

### PAH8 profiling in marine products

**Fig. 1:**
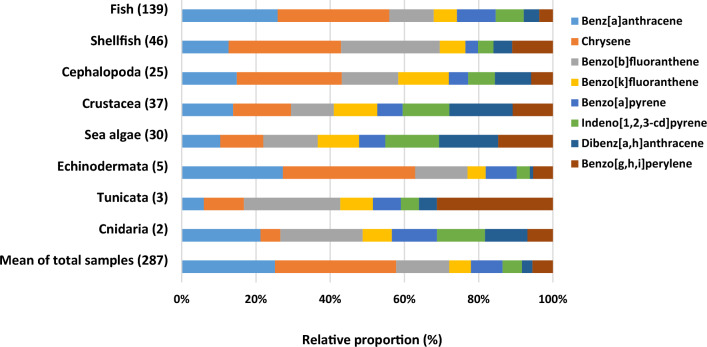
Relative proportions of mean concentrations of 8 polycyclic aromatic hydrocarbons (PAH8) in 287 marine product subsamples under medium-bound state

The relative proportions of PAH8 in the medium-bound state within eight categories are described in Fig. 1. Chrysene was a primary component accounting for 33% of an amount of PAH8 in total marine products, followed by B[a]A at 25%. In PAH8, D[a,h]A has the lowest percentage at 3%, followed by I[c,d]P at 5%. These distributions showed the similar tendency in which EFSA (2008) reported that chrysene was a dominant element showing 33% followed by B[a]A at 20%, while D[a,h]A made up the lowest rate at 2% followed by B[k]F at 6% (EFSA, 2008). Chrysene showed the highest contribution in French TDS as well, followed by B[b]F (Veyrand et al., 2013).

**Fig. 2:**
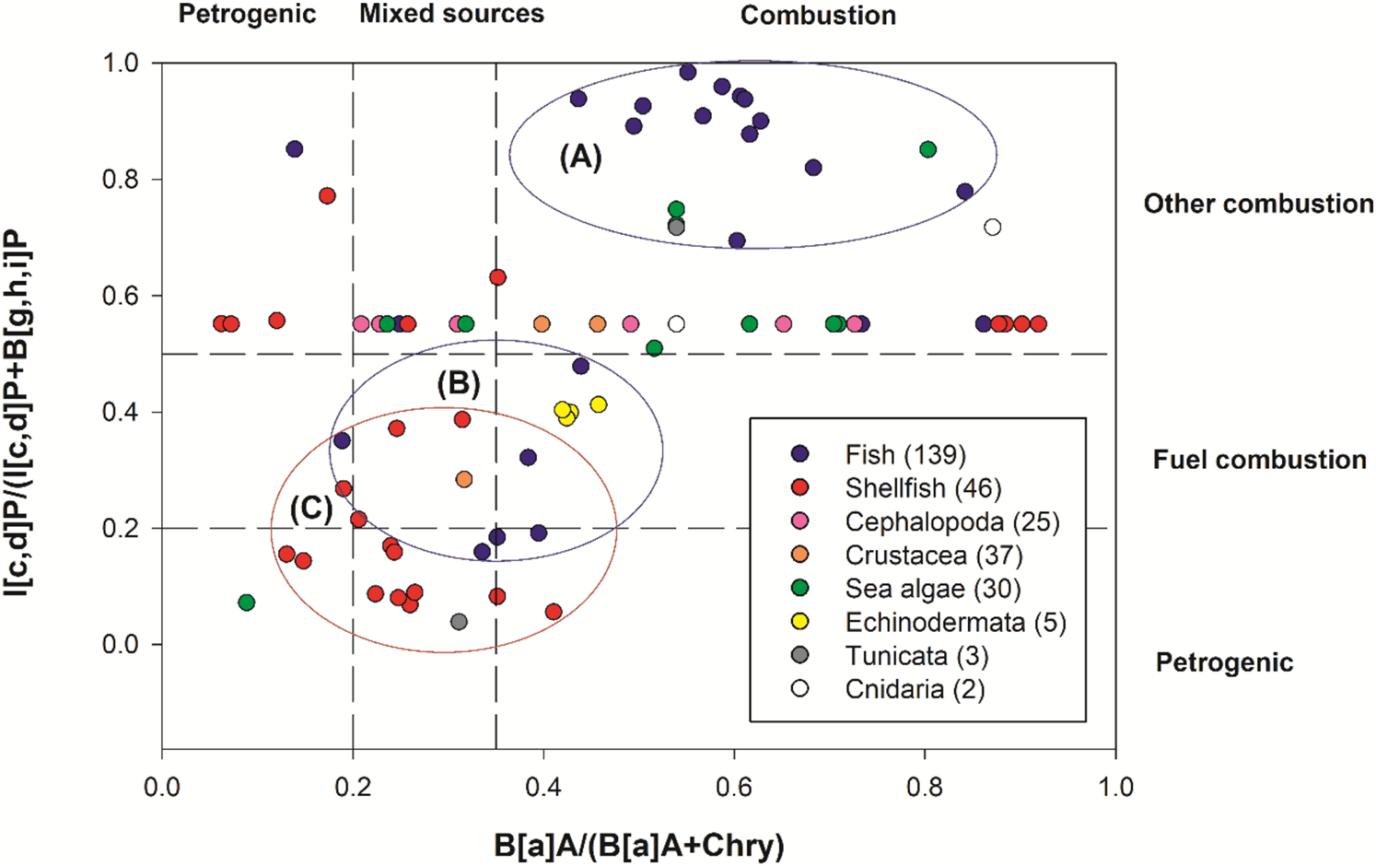
B[a]A/(B[a]A + Chry) ratio plotted against the I[c,d]P/(I[c,d]P + B[g,h,i]P ratio on a scatter plot for marine product subsamples. Reference ranges for diagnostic ratios were applied (Yan et al., 2005). B[a]A stands for Benz[a]pyrene, whereas Chry, Indeno[1,2,3-cd]pyrene, and B[g,h,i]P stand for Benzo[g,h,i]perylene

In Fig. 2, the ratios of B[a]A/(B[a]A + Chry) and I[c,d]P/(I[c,d]P + B[g,h,i]P were displayed. These ratios were employed as a marker to determine the origin of PAHs (Yan et al., 2005; Yunker et al., 2002). Medium bound was applied to obtain the ratios in the present study. When PAH8 were found in fish subsamples above LODs, they typically underwent heating processes like grilling or drying (Supplementary Table 2). Most of the dots in the plot's zone (A) are grilled fish, whereas those on the plot's zone (B) are often dried fish (Fig. 2). These patterns, specifically, demonstrate that whereas dried fish contains petrogenic PAHs from the environment, grilled fish contains PAHs produced by burning. In general, it has been noted that grilled food is one of the major contributors to PAH consumption (EFSA, 2008).

In the shellfish category, however, PAH8 concentrations were frequently above LODs in raw materials, and the variance of PAH8 levels depending on the heating methods was smaller than in the fish category (Supplementary Table 2). According to zone (C) in the scatter plot, the PAH8 in the shellfish samples might be derived from petrogenic origin rather than from incomplete combustion (Fig. 2). Fernando et al (2019) reported that shellfish products were contaminated from spilt crude oil in sea water (Fernando et al., 2019). In this research, cephalopods had a slightly larger B[a]A/(B[a]A + Chry) ratio (0.3) than the 0.2 from the Second French TDS (Veyrand et al., 2013) based on mean distributions of B[a]A and Chry.

### Dietary exposure and risk assessment

To calculate daily dietary exposure, the daily food intake rate was multiplied by the total PAH8 concentration (∑PAH8) value or the TEQ_B[a]P_ value of PAH8 in the marine products using different cooking methods, and then divided the result by body weight. The outcomes are represented in Table 3 with both LB and UB values. On average, South Koreans are exposed to 1.278 ng per kg of body weight per day (ng/kg bw/d) of ∑PAH8 and 0.399 ng/kg bw/d of TEQB[a]P from total marine products under the LB scenario. Under the UB scenario, the average exposure is 2.164 ng/kg bw/d for ∑PAH8 and 1.347 ng/kg bw/d for TEQB[a]P. The 1–2 age group's mean dietary exposure to PAH8 from total marine products utilizing the UB scenario had the greatest mean daily exposure (3.212 ng/kg bw/d for ∑PAH8 and 2.071 ng/kg bw/d for TEQ_B[a]P_) due to their lowest body weights across all age groups.

**Table 3:** Mean dietary exposure to (a) total PAH8 concentration (∑PAH8) and (b) total B[a]P toxic equivalent quantity (TEQ_B[a]p_) of PAH8, with contributions from marine products, for the total consumer population and different age groups, with lower bound (LB) and upper bound (UB) estimates

	1–2 years (n = 519)				3–6 years (n = 1,062)				7–12 years (n = 1,601)				13–19 years (n = 1,629)				20–64 years (n = 11,592)				over 64 years (n = 4,268)				Total population (n = 20,671)			
	Mean dietary exposure		Contribution		Mean dietary exposure		Contribution		Mean dietary exposure		Contribution		Mean dietary exposure		Contribution		Mean dietary exposure		Contribution		Mean dietary exposure		Contribution		Mean dietary exposure		Contribution	
	(ng/kg bw/day)		(%)		(ng/kg bw/day)		(%)		(ng/kg bw/day)		(%)		(ng/kg bw/day)		(%)		(ng/kg bw/day)		(%)		(ng/kg bw/day)		(%)		(ng/kg bw/day)		(%)	
a)																												
Category	LB	UB	LB	UB	LB	UB	LB	UB	LB	UB	LB	UB	LB	UB	LB	UB	LB	UB	LB	UB	LB	UB	LB	UB	LB	UB	LB	UB
(Number of composite samples)																												
Fish (55)	1.467	1.905	84.7	59.3	1.486	1.857	88.8	65.6	0.791	1.089	73.9	56.5	0.466	0.659	74.8	57.2	0.866	1.235	60.1	52.3	0.562	0.873	77.6	55.3	0.805	1.152	63.0	53.2
Shellfish (17)	0.117	0.166	6.8	5.2	0.066	0.104	3.9	3.7	0.088	0.128	8.2	6.7	0.060	0.089	9.6	7.7	0.123	0.179	8.5	7.6	0.084	0.117	11.6	7.4	0.111	0.162	8.7	7.5
Cephalopoda (8)	0.129	0.187	7.5	5.8	0.093	0.222	5.5	7.8	0.096	0.184	9.0	9.6	0.055	0.134	8.8	11.6	0.078	0.181	5.4	7.7	0.014	0.066	1.9	4.2	0.069	0.164	5.4	7.6
Crustacea (8)	0.005	0.103	0.3	3.2	0.006	0.116	0.4	4.1	0.006	0.088	0.5	4.6	0.002	0.060	0.3	5.2	0.004	0.067	0.3	2.8	0.004	0.041	0.5	2.6	0.004	0.065	0.3	3.0
Sea algae (16)	0.012	0.850	0.7	26.5	0.014	0.523	0.9	18.5	0.009	0.354	0.8	18.4	0.004	0.170	0.6	14.8	0.005	0.327	0.4	13.9	0.004	0.421	0.6	26.7	0.005	0.333	0.4	15.4
Echinodermata (2)	0.000	0.000	0.0	0.0	0.000	0.000	0.0	0.0	0.076	0.076	7.1	4.0	0.021	0.022	3.4	1.9	0.350	0.350	24.3	14.8	0.048	0.048	6.6	3.1	0.269	0.270	21.1	12.5
Tunicata (2)	0.000	0.001	0.0	0.0	0.009	0.010	0.5	0.4	0.005	0.007	0.5	0.3	0.015	0.018	2.5	1.6	0.015	0.019	1.0	0.8	0.008	0.011	1.2	0.7	0.014	0.017	1.1	0.8
Cnidaria (1)	0.000	0.000	0.0	0.0	0.000	0.000	0.0	0.0	0.000	0.000	0.0	0.0	0.000	0.000	0.0	0.0	0.001	0.002	0.1	0.1	0.000	0.001	0.0	0.1	0.001	0.002	0.0	0.1
Total marine products (109)	1.731	3.212	100.0	100.0	1.673	2.833	100.0	100.0	1.071	1.926	100.0	100.0	0.623	1.151	100.0	100.0	1.440	2.360	100.0	100.0	0.724	1.578	100.0	100.0	1.278	2.164	100.0	100.0
b)																												
Category	LB	UB	LB	UB	LB	UB	LB	UB	LB	UB	LB	UB	LB	UB	LB	UB	LB	UB	LB	UB	LB	UB	LB	UB	LB	UB	LB	UB
(Number of composite samples)																												
Fish (55)	0.538	1.098	96.6	53.0	0.580	1.033	97.3	55.9	0.294	0.652	91.0	52.2	0.294	0.652	91.0	52.2	0.362	0.787	81.7	54.7	0.243	0.603	93.2	53.9	0.334	0.735	83.7	54.6
Shellfish (17)	0.006	0.083	1.2	4.0	0.005	0.062	0.8	3.4	0.006	0.069	1.9	5.5	0.006	0.069	1.9	5.5	0.009	0.100	2.1	7.0	0.006	0.058	2.2	5.2	0.008	0.085	2.1	6.3
Cephalopoda (8)	0.009	0.099	1.7	4.8	0.007	0.183	1.1	9.9	0.007	0.130	2.2	10.4	0.007	0.130	2.2	10.4	0.006	0.143	1.3	9.9	0.001	0.068	0.4	6.1	0.005	0.131	1.2	9.7
Crustacea (8)	0.000	0.113	0.0	5.5	0.000	0.127	0.1	6.9	0.000	0.096	0.1	7.7	0.000	0.096	0.1	7.7	0.000	0.073	0.0	5.1	0.000	0.044	0.1	3.9	0.000	0.071	0.0	5.3
Sea algae (16)	0.003	0.677	0.5	32.7	0.003	0.437	0.5	23.7	0.001	0.284	0.5	22.8	0.001	0.284	0.5	22.8	0.001	0.262	0.2	18.2	0.001	0.331	0.4	29.6	0.001	0.266	0.3	19.8
Echinodermata (2)	0.000	0.000	0.0	0.0	0.000	0.000	0.0	0.0	0.014	0.014	4.2	1.1	0.014	0.014	4.2	1.1	0.063	0.064	14.2	4.4	0.009	0.009	3.3	0.8	0.048	0.049	12.2	3.6
Tunicata (2)	0.000	0.001	0.0	0.1	0.001	0.005	0.2	0.2	0.001	0.003	0.2	0.3	0.001	0.003	0.2	0.3	0.002	0.009	0.4	0.6	0.001	0.005	0.4	0.4	0.002	0.008	0.4	0.6
Cnidaria (1)	0.000	0.000	0.0	0.0	0.000	0.000	0.0	0.0	0.000	0.000	0.0	0.0	0.000	0.000	0.0	0.0	0.000	0.002	0.0	0.1	0.000	0.001	0.0	0.1	0.000	0.002	0.0	0.1
Total marine products (109)	0.557	2.071	100.0	100.0	0.596	1.848	100.0	100.0	0.323	1.248	100.0	100.0	0.323	1.248	100.0	100.0	0.443	1.438	100.0	100.0	0.261	1.119	100.0	100.0	0.399	1.347	100.0	100.0

The results of below LOD are substituted for zero at the LB, while the results are replaced by LOD at the UB (EFSA, 2010; WHO/IPCS, 2009)

In this study, dietary exposure to PAHs from fish is higher than from shellfish, cephalopods and crustaceans. The average dietary intake for the fish category was found to be 0.805 ng/kg bw/d for ∑PAH8 and 0.334 ng/kg bw/d for TEQ_B[a]P_ in the LB scenario, while in the UB scenario, it was 1.152 ng/kg bw/d for ∑PAH8 and 0.735 ng/kg bw/d for TEQ_B[a]P_ for the total population, whereas the overall dietary exposure for the shellfish, cephalopods, and crustaceans was 0.184 ng/kg bw/d for ∑PAH8 and 0.013 ng/kg bw/d for TEQ_B[a]P_ under the LB scenario, and 0.391 ng/kg bw/d for ∑PAH8 and 0.287 ng/kg bw/d for TEQ_B[a]P_ under the UB scenario for total population (Table 3). However, it is important to note that shellfish, cephalopods, and crustaceans constituted the primary contributors to PAH exposure during the Second French TDS (Veyrand et al., 2013), which is believed to be due to varying consumption habits among country. In their investigation, the average dietary exposure from fish was 0.03 ng/kg bw/d for adults and 0.07 ng/kg bw/d for children, compared to 0.193 ng/kg bw/d for adults and 0.098 ng/kg bw/d for children from mollusks and crustaceans. As seen in Table 3, fish products were the highest contributor to dietary exposure in this study, ranging from 63.0% for ∑PAH8 (83.7% for TEQ_B[a]P_) in the total population to 88.8% for ∑PAH8 (97.3% for TEQ_B[a]P_) in the 3–6 year old population using the LB value, and from 53.2% for ∑PAH8 (54.6% for TEQ_B[a]P_) in the total population to 65.6% for ∑PAH8 (55.9% for TEQ_B[a]P_) in the 3–6 year old population using the UB value. Sea algae was the next major contributor, ranging from 15.4% for ∑PAH8 (19.8% for TEQ_B[a]P_) in the total population to for 18.5% ∑PAH8 (23.7% for TEQ_B[a]P_) in 3–6 years population under the UB scenario, while its contribution was smaller under the LB scenario. The high consumption level of sea algae appears to result from its significant contribution, despite its low contamination level. Particularly in the 20–64 age group under the LB scenario, Echinodermata, including dried sea cucumber, also made a considerable contribution of 24.3% for ∑PAH8 (14.2% for TEQ_B[a]P_). The marine subsample in Supplementary Table 1 and 2 with the highest dietary exposure level was dried sea cucumber, which the adult group consumed more than the other groups.

The MOE approach was used for the risk assessment. According to the findings of this study, independent of age groups or marine products, all MOEs were above 10,000 (Table 4). The calculated MOEs were about 1,055,476,358 for ∑PAH8 (6,469,460,509 for TEQ_B[a]P_) at the LB scenario and 346,669,319 for ∑PAH8 (407,307,792) for TEQ_B[a]P_) at the UB scenario for the total population. It's important to note that the MOEs for the calculated equivalent B[a]P, as determined by TEQ (TEQ_B[a]P_), in the case of the other 7 PAHs, ranged from 1.2 times higher in the UB scenario to 6.1 times higher in the LB scenario compared to those of ∑PAH8. All MOEs exceeded 1.0 × 10^4^, indicating that the risk assessment results indicate a low level of concern from a public perspective regarding health risks associated with dietary exposure to PAH8 from marine products. In the 1–2 age group under the UB scenario, the risk of PAH8 from fish diet had the lowest MOE value (257,240 for ∑PAH8 and 446,434 for TEQ_B[a]P_), followed by the risk of PAH8 through sea algae (MOE value: 576,548 for ∑PAH8 and 724,207 for TEQ_B[a]P_).

**Table 4:** Margin of exposure (MOE) of a) total PAH8 concentration (∑PAH8) and b) total B[a]P toxic equivalent quantity (TEQ_B[a]p_) of PAH8, with contributions from marine products, for the total consumer population and different age groups, with lower bound (LB) and upper bound (UB) estimates

Category	1–2 years (n = 519)		3–6 years (n = 1062)		7–12 years (n = 1601)		13–19 years (n = 1629)		20–64 years (n = 11,592)		over 64 years (n = 4268)		Total population (n = 20,671)	
(Number of composite samples)	LB	UB	LB	UB	LB	UB	LB	UB	LB	UB	LB	UB	LB	UB
a)														
Fish (55)	334,126	257,240	329,672	263,828	619,093	449,885	1,051,318	744,090	566,051	396,746	872,216	561,154	608,627	425,384
Shellfish (17)	4,173,301	2,948,542	7,478,775	4,722,478	5,589,635	3,822,012	8,217,150	5,511,352	3,998,845	2,730,755	5,835,694	4,201,324	4,413,599	3,025,218
Cephalopoda (8)	3,794,026	2,616,220	5,293,790	2,205,153	5,108,335	2,656,666	8,889,355	3,656,622	6,277,911	2,703,296	35,798,002	7,379,119	7,054,954	2,978,939
Crustacea (8)	94,098,922	4,771,464	77,683,847	4,219,418	84,572,057	5,571,220	313,668,411	8,208,975	135,702,943	7,360,908	123,900,514	11,871,437	135,341,634	7,565,106
Sea algae (16)	39,768,295	576,548	34,393,293	936,354	54,995,892	1,386,110	130,885,860	2,881,249	97,036,196	1,498,867	118,496,348	1,163,862	95,388,260	1,472,543
Echinodermata (2)	–	–	–	–	6,445,122	6,433,294	22,903,645	22,647,957	1,401,658	1,399,306	10,230,093	10,164,192	1,821,130	1,817,747
Tunicata (2)	1,649,994,173	347,735,179	57,460,091	46,883,430	99,486,485	75,014,965	31,639,822	27,095,451	33,139,024	26,196,820	58,251,076	45,630,811	35,877,873	28,543,013
Cnidaria (1)	–	–	–	–	9,316,679,023	3,616,709,611	4,707,911,175	1,827,598,391	624,141,042	242,289,865	1,542,127,897	598,649,881	774,970,282	300,841,368
Total marine products (109)	1,792,162,843	358,905,194	182,639,467	59,230,660	9,573,495,642	3,712,043,762	5,225,166,737	1,898,344,087	902,263,669	284,576,563	1,895,511,840	679,621,779	1,055,476,358	346,669,319
b)														
Fish (55)	910,244	446,434	844,739	474,122	1,667,605	751,356	2,766,079	1,206,274	1,354,396	622,877	2,016,766	812,546	1,467,855	666,886
Shellfish (17)	76,293,931	5,884,162	100,892,860	7,858,807	80,115,752	7,115,484	125,170,607	9,945,102	51,909,404	4,900,896	83,707,628	8,376,380	58,483,416	5,755,430
Cephalopoda (8)	53,148,375	4,951,244	73,248,030	2,678,672	69,919,626	3,767,841	125,452,128	4,489,832	88,277,852	3,436,359	446,805,458	7,235,757	98,751,030	3,738,716
Crustacea (8)	1,770,251,200	4,339,809	1,461,439,931	3,848,072	1,591,025,493	5,125,266	5,900,937,719	7,332,007	2,552,933,572	6,712,720	2,330,898,461	11,173,842	2,546,136,377	6,907,488
Sea algae (16)	179,469,856	724,207	156,997,074	1,120,714	336,997,268	1,723,959	693,764,875	3,533,473	491,833,900	1,871,167	536,103,140	1,480,177	482,062,531	1,840,611
Echinodermata (2)	–	–	–	–	35,763,094	35,292,123	127,177,853	117,539,177	7,777,516	7,683,768	56,784,840	54,231,720	10,105,218	9,970,520
Tunicata (2)	16,499,941,725	334,063,204	451,146,740	107,479,626	785,944,268	150,611,606	247,544,766	68,457,950	260,817,831	56,751,502	458,784,952	97,342,423	282,235,083	62,517,277
Cnidaria (1)	–	–	–	–	35,948,359,927	3,797,862,795	18,165,451,982	1,919,138,853	2,408,245,125	254,425,642	5,950,292,862	628,634,962	2,990,218,999	315,909,864
Total marine products (109)	18,580,015,331	350,409,060	2,244,569,374	123,460,013	38,849,793,033	4,002,250,430	25,388,266,009	2,131,642,668	5,863,149,596	336,404,931	9,865,394,107	809,287,807	6,469,460,509	407,306,792

Individual MOE values for composite samples are further included in Supplementary Tables 1 and 2. Considering the total population based on KNHANES (MOHW, 2013), the MOE for all individual samples was above 10,000. When considering only those people in the consumption group who consumed the specific food samples listed in the KNHANES under the UB scenario, dried sea cucumber had the lowest MOE value, with 6,201 for ∑PAH8 and 38,651 for TEQ_B[a]P_. Salted mackerel had the second lowest MOE values, with 36,481 for ∑PAH8 and 87,244 for TEQ_B[a]P_. This is attributed to their elevated levels of PAH8 and substantial consumption rates. On the other hand, katsuobushi showed relatively high MOE value (758,956 for ∑PAH8 and 4,934,786 for TEQ_B[a]P_), even though it contained high PAH8 level. Katsuobushi is typically used as a stock ingredient or spice in Korean cuisine, resulting in modest daily intakes. Another study also reported that Katsuobushi had comparably low daily exposure to PAHs because to its low consumption rate (Lee et al., 2018).

In conclusion, this study found that fish is the primary source of PAH exposure in marine products, and that grilling, smoking, and drying processes increase PAH levels in food. The estimated risk of exposure to eight PAHs using TEFs may be an overestimation. It is advisable for many studies using TEFs to consider using the total concentration and the toxicological values for PAH mixtures. Following a risk assessment using a MOE approach in a TDS, PAH8 exposure from marine products is considered low for the South Korean population. The findings of this study can serve as a foundation for developing strategies to reduce PAH levels in food and to identify the most effective ways for consuming marine products.

### Supplementary Information

Below is the link to the electronic supplementary material.

Supplementary file1 (DOCX 97 KB)

## Data Availability

The data that support the findings of this study are available on request from the corresponding author. The data are not publicly available due to privacy or ethical restrictions.
