# Supplementary material for: Evaluation of polycyclic aromatic hydrocarbon contents in marine products in South Korea and risk assessment using the total diet study
Source: Food Sci Biotechnol. 2024 Jan 9;33(10):2377–90. doi: 10.1007/s10068-023-01491-y (PMC11319570; doi:10.1007/s10068-023-01491-y)
Supplement: Supplementary file 1 — Supplementary file1 (DOCX 97 KB) [file 10068_2023_1491_MOESM1_ESM.docx]

**Supplementary Table 1. Total PAH8 concentration (****∑PAH8), dietary exposure, and margin of exposure (MOE) of 109 marine products samples under lower bound (LB) and upper bound (UB) by total population and consumed group**

|  |  |  |  |  |  | Total population | | | | Consumption group | | | |
| --- | --- | --- | --- | --- | --- | --- | --- | --- | --- | --- | --- | --- | --- |
|  |  |  |  | PAH8 (μg/kg) | | Dietary exposure (ng/kg/d) | | MOE | | Dietary exposure  (ng/kg/d) | | MOE | |
| Category | Composite sample | State of composite sample | Cooking method | LB | UB | LB | UB | LB | UB | LB | UB | LB | UB |
| Fish | Mackerel | Raw | None, Boiling, Grilling, and Pan-frying | 4.673 | 5.351 | 0.163 | 0.187 | 3005812 | 2625362 | 3.646 | 4.174 | 134411 | 117398 |
|  | Mackerel | Salted | None, Grilling, and Pan-frying | 12.349 | 12.928 | 0.464 | 0.486 | 1055753 | 1008496 | 12.831 | 13.432 | 38190 | 36481 |
|  | Japanese spanish mackerel | Raw | None, Boiling, and Grilling | 0.437 | 1.109 | 0.005 | 0.013 | 93405697 | 36795719 | 0.480 | 1.219 | 1020505 | 402012 |
|  | Atka mackerel | Raw | None, Boiling, Grilling, and Airfrying | 0.709 | 1.430 | 0.004 | 0.008 | 125124366 | 62035429 | 0.808 | 1.630 | 606294 | 300594 |
|  | Horse mackerel | Raw | None and Boiling | 0.000 | 0.868 | 0.000 | 0.000 | - | - | 0.000 | 0.000 | - | - |
|  | Brown sole fish | Raw | None, Boiling, Grilling, and Airfrying | 0.000 | 0.868 | 0.000 | 0.009 | - | 56691833 | 0.000 | 0.757 | - | 647024 |
|  | Olive flounder | Raw | None and Boiling | 0.000 | 0.868 | 0.000 | 0.020 | - | 24229923 | 0.000 | 1.093 | - | 448272 |
|  | Finespotted flounder | Raw | None, Boiling, and Grilling | 0.735 | 1.407 | 0.001 | 0.003 | 358690363 | 187423058 | 1.244 | 2.380 | 393940 | 205842 |
|  | Summer flounder | Raw | None, Boiling, and Grilling | 1.585 | 2.257 | 0.005 | 0.007 | 102715292 | 72129874 | 1.313 | 1.870 | 373191 | 262066 |
|  | Alaska pollock | Raw | None and Boiling | 0.000 | 0.868 | 0.000 | 0.005 | - | 106854192 | 0.000 | 0.961 | - | 509821 |
|  | Alaska pollock | Frozen | None, Pan-frying, and Airfrying | 0.000 | 0.868 | 0.000 | 0.021 | - | 23145554 | 0.000 | 0.723 | - | 678155 |
|  | Alaska pollock | Semi-dried | None, Boiling, and Airfrying | 0.000 | 0.868 | 0.000 | 0.010 | - | 46693469 | 0.000 | 0.903 | - | 542494 |
|  | Alaska pollock | Dried | None, Boiling, and Grilling | 1.727 | 2.172 | 0.026 | 0.032 | 19121558 | 15204825 | 0.646 | 0.812 | 758597 | 603211 |
|  | Alaska pollock roe | Raw | None and Boiling | 0.000 | 0.868 | 0.000 | 0.001 | - | 617930457 | 0.000 | 0.494 | - | 991039 |
|  | Alaska pollock roe | Salted | None | 0.000 | 0.868 | 0.000 | 0.001 | - | 437092541 | 0.000 | 0.188 | - | 2600990 |
|  | Alaska pollack tripe | Salted | None | 0.104 | 0.881 | 0.000 | 0.001 | 6689068921 | 788916922 | 0.012 | 0.105 | 39403181 | 4647259 |
|  | Pacific cod | Raw | None, Boiling, and Airfrying | 0.000 | 0.868 | 0.000 | 0.007 | - | 69620509 | 0.000 | 0.781 | - | 627495 |
|  | Pacific cod | Dried | None | 0.430 | 1.111 | 0.001 | 0.003 | 406312687 | 157183038 | 0.389 | 1.005 | 1260746 | 487723 |
|  | Pacific cod roe | Raw | None and Boiling | 0.000 | 0.868 | 0.000 | 0.001 | - | 732009926 | 0.000 | 0.252 | - | 1943359 |
|  | Pacific cod roe and milt | Raw | None | 0.000 | 0.868 | 0.000 | 0.001 | - | 843201307 | 0.000 | 0.064 | - | 7686163 |
|  | Brown croaker | Raw | None, Boiling, and Grilling | 0.318 | 1.018 | 0.001 | 0.002 | 931024280 | 291323316 | 0.300 | 0.959 | 1633582 | 511158 |
|  | Yellow croaker | Raw | None, Boiling, Grilling, Pan-frying, and Steaming | 0.902 | 1.653 | 0.017 | 0.032 | 28291309 | 15447118 | 0.540 | 0.989 | 907355 | 495418 |
|  | Yellow croaker | Salt-dried | None, Boiling, Steaming, and Grilling | 0.593 | 1.314 | 0.007 | 0.015 | 74686469 | 33698651 | 0.534 | 1.184 | 917571 | 414009 |
|  | Silver pomfret | Raw | None, Boiling, Airfrying, and Grilling | 0.000 | 0.868 | 0.000 | 0.001 | - | 763909441 | 0.000 | 0.479 | - | 1022966 |
|  | Japanese icefish | Dried | None, Grilling, and Stir-frying | 0.509 | 1.126 | 0.002 | 0.004 | 256273001 | 115826116 | 0.136 | 0.301 | 3606569 | 1630039 |
|  | Anchovy | Raw | None and Boiling | 0.000 | 0.868 | 0.000 | 0.003 | - | 182201595 | 0.000 | 0.522 | - | 938318 |
|  | Anchovy | Salted | None | 0.000 | 0.868 | 0.000 | 0.002 | - | 320871403 | 0.000 | 0.062 | - | 7907139 |
|  | Anchovy  (Large size) | Dried | None and Steeping | 0.000 | 0.868 | 0.000 | 0.009 | - | 55594144 | 0.000 | 0.037 | - | 13243122 |
|  | Anchovy (Medium size) | Dried | None and Stir-frying | 0.642 | 1.028 | 0.005 | 0.008 | 96819276 | 60504397 | 0.052 | 0.084 | 9342704 | 5838452 |
|  | Anchovy (Small size) | Dried | None and Stir-frying | 0.000 | 0.868 | 0.000 | 0.008 | - | 58319824 | 0.000 | 0.070 | - | 6998771 |
|  | Tuna | Raw | None | 0.000 | 0.868 | 0.000 | 0.013 | - | 38234935 | 0.000 | 0.613 | - | 799157 |
|  | Tuna | Canned | None, Boiling, and Stir-frying | 0.000 | 0.868 | 0.000 | 0.005 | - | 108137830 | 0.000 | 0.647 | - | 757743 |
|  | Skipjack tuna | Canned | None, Boiling, and Stir-frying | 0.047 | 0.875 | 0.000 | 0.005 | 1730301974 | 93587351 | 0.021 | 0.384 | 23616320 | 1277343 |
|  | Katsuobushi | Smoked | None and Steeping | 110.234 | 110.668 | 0.010 | 0.010 | 50434863 | 50237075 | 0.643 | 0.646 | 761944 | 758956 |
|  | Katsuobushi stock | Smoked and steeped | None | 0.000 | 0.868 | 0.000 | 0.005 | - | 93452446 | 0.000 | 0.203 | - | 2415822 |
|  | Cutlassfish | Raw | None, Boiling, and Pan-frying | 0.000 | 0.868 | 0.000 | 0.015 | - | 32370932 | 0.000 | 0.635 | - | 771329 |
|  | Pacific saury | Raw | None, Boiling, and Pan-frying | 0.000 | 0.868 | 0.000 | 0.009 | - | 56166023 | 0.000 | 0.459 | - | 1068549 |
|  | Pacific saury | Canned | None and Boiling | 0.000 | 0.868 | 0.000 | 0.004 | - | 122766132 | 0.000 | 0.280 | - | 1752298 |
|  | Sailfin sandfish | Raw | None and Boiling | 0.000 | 0.868 | 0.000 | 0.003 | - | 171241396 | 0.000 | 0.870 | - | 563333 |
|  | Amur catfish | Raw | None and Boiling | 0.000 | 0.868 | 0.000 | 0.004 | - | 134734837 | 0.000 | 1.547 | - | 316818 |
|  | Pond loach | Raw | None and Boiling | 0.000 | 0.868 | 0.000 | 0.013 | - | 37159937 | 0.000 | 1.295 | - | 378504 |
|  | Japanese amberjack | Raw | None, Boiling, Grilling, and Airfrying | 0.000 | 0.868 | 0.000 | 0.000 | - | 1106526632 | 0.000 | 1.023 | - | 478910 |
|  | Pufferfish | Raw | None, Boiling, and Airfrying | 0.000 | 0.868 | 0.000 | 0.004 | - | 134789363 | 0.000 | 1.519 | - | 322581 |
|  | Dark banded rockfish | Raw | None, Boiling, and Grilling | 0.000 | 0.868 | 0.000 | 0.013 | - | 37930135 | 0.000 | 1.063 | - | 460768 |
|  | Flathead mullet | Raw | None and Boiling | 0.000 | 0.868 | 0.000 | 0.001 | - | 380210635 | 0.000 | 1.147 | - | 427170 |
|  | Blackmouth angler | Raw | None and Boiling | 0.000 | 0.868 | 0.000 | 0.010 | - | 50099957 | 0.000 | 1.692 | - | 289569 |
|  | Chum salmon | Raw | None, Airfrying, and Pan-frying | 0.000 | 0.868 | 0.000 | 0.006 | - | 81294732 | 0.000 | 0.612 | - | 800092 |
|  | Freshwater eel | Raw | None, Boiling, and Grilling | 2.578 | 3.192 | 0.064 | 0.079 | 7682228 | 6205194 | 3.912 | 4.843 | 125250 | 101169 |
|  | Sea eel | Raw | None and Boiling | 0.000 | 0.868 | 0.000 | 0.004 | - | 112029773 | 0.000 | 0.975 | - | 502489 |
|  | Gizzard shad | Raw | None, Boiling, and Grilling | 0.085 | 0.921 | 0.001 | 0.007 | 770502319 | 71084586 | 0.143 | 1.551 | 3425255 | 316005 |
|  | Filefish | Dried | None, Boiling, and Grilling | 6.150 | 6.430 | 0.030 | 0.031 | 16361456 | 15649006 | 2.380 | 2.488 | 205891 | 196925 |
|  | Ray | Raw | None and Boiling | 0.000 | 0.868 | 0.000 | 0.001 | - | 634408602 | 0.000 | 0.700 | - | 699880 |
|  | Skate | Fermented | None, Steaming, and Boiling | 0.000 | 0.868 | 0.000 | 0.011 | - | 44598891 | 0.000 | 1.134 | - | 432204 |
|  | Flying fish roe | Raw | None | 0.000 | 0.868 | 0.000 | 0.000 | - | 1176906417 | 0.000 | 0.140 | - | 3500268 |
|  | Fish sauce | Salted | None, Boiling, and Stir-frying | 0.000 | 0.765 | 0.000 | 0.002 | - | 222429957 | 0.000 | 0.016 | - | 31142027 |
| Shellfish | Clam | Raw | None, Boiling, and Steaming | 0.637 | 1.318 | 0.002 | 0.003 | 295374779 | 142781469 | 0.139 | 0.287 | 3526753 | 1704800 |
|  | Manila clam | Raw | None and Boiling | 0.734 | 1.380 | 0.016 | 0.030 | 30413903 | 16175677 | 0.138 | 0.260 | 3542645 | 1884161 |
|  | Manila clam | Salted | None | 2.469 | 2.644 | 0.001 | 0.002 | 328022227 | 306309148 | 0.341 | 0.366 | 1435326 | 1340316 |
|  | Marsh clam | Raw | None and Boiling | 3.490 | 3.914 | 0.002 | 0.002 | 323616754 | 288556174 | 0.500 | 0.561 | 980170 | 873978 |
|  | Scallop | Raw | None, Boiling, and Grilling | 1.456 | 1.854 | 0.010 | 0.012 | 51252295 | 40247959 | 2.409 | 3.068 | 203413 | 159739 |
|  | Oyster | Raw | None, Boiling, and Pan-frying | 3.114 | 3.608 | 0.045 | 0.052 | 10822151 | 9340343 | 1.661 | 1.925 | 295000 | 254607 |
|  | Oyster | Salted | None | 2.753 | 3.162 | 0.002 | 0.003 | 200058421 | 174177309 | 1.284 | 1.475 | 381537 | 332178 |
|  | Blood cockle | Raw | None, Boiling, and Parboiling | 0.250 | 0.827 | 0.002 | 0.007 | 244973618 | 74175016 | 0.303 | 1.000 | 1618738 | 490134 |
|  | Pen shell | Raw | None, Boiling, and Grilling | 0.455 | 1.232 | 0.001 | 0.003 | 523936511 | 193369854 | 0.384 | 1.040 | 1276732 | 471205 |
|  | Red shell | Raw | None and Boiling | 1.640 | 1.964 | 0.002 | 0.002 | 272450505 | 227563494 | 0.798 | 0.956 | 613911 | 512768 |
|  | Mussel | Raw | None, Boiling, Pan-frying, and Stir-frying | 1.631 | 2.149 | 0.018 | 0.024 | 27322868 | 20735635 | 0.635 | 0.837 | 771690 | 585644 |
|  | Mussel | Boiled-dried | None, Stir-frying after soaking, Pan-frying after soaking, and Boiling | 6.047 | 6.421 | 0.010 | 0.011 | 47757232 | 44975757 | 0.811 | 0.861 | 604331 | 569133 |
|  | Whelk | Raw | None, Boiling, and Parboiling | 0.025 | 0.863 | 0.000 | 0.001 | 17881689263 | 516422409 | 0.022 | 0.756 | 22437245 | 647987 |
|  | Whelk | Canned | None | 0.000 | 0.868 | 0.000 | 0.005 | - | 106037732 | 0.000 | 0.794 | - | 616755 |
|  | Abalone | Raw | None, Boiling, Pan-frying, and Steaming | 0.000 | 0.868 | 0.000 | 0.004 | - | 110836777 | 0.000 | 0.223 | - | 2197677 |
|  | Freshwater snail | Raw | None, Boiling, and Parboiling | 0.000 | 0.868 | 0.000 | 0.001 | - | 507720299 | 0.000 | 0.093 | - | 5264178 |
|  | Conch | Raw | None, Parboiling, Boiling after parboiling, and Stir-frying after parboiling | 0.000 | 0.868 | 0.000 | 0.001 | - | 674219668 | 0.000 | 0.151 | - | 3234547 |
| Cephalopoda | Squid | Raw | None, Boiling, Pan-frying, Airfrying, Stir-frying, and Parboiling | 0.133 | 0.873 | 0.009 | 0.060 | 53675870 | 8180227 | 0.069 | 0.452 | 7112484 | 1083946 |
|  | Squid | Dried | None and Grilling | 3.048 | 3.492 | 0.057 | 0.066 | 8550819 | 7463721 | 1.822 | 2.088 | 268900 | 234714 |
|  | Squid | Salted | None | 0.095 | 0.872 | 0.000 | 0.002 | 1968977619 | 214454823 | 0.011 | 0.098 | 46040889 | 5014628 |
|  | Cuttlefish | Raw | None, Stir-frying, Pan-frying, and Blanching | 0.060 | 0.898 | 0.000 | 0.001 | 7307268716 | 486438818 | 0.023 | 0.349 | 21078782 | 1403197 |
|  | Octopus | Raw | None, Boiling, and Parboiling | 0.000 | 0.868 | 0.000 | 0.005 | - | 92620833 | 0.000 | 0.328 | - | 1493777 |
|  | Octopus | Dried | None and Grilling | 2.856 | 3.265 | 0.003 | 0.003 | 181112471 | 158421664 | 1.366 | 1.561 | 358754 | 313807 |
|  | Small octopus | Raw | None, Boiling, and Stir-frying | 0.000 | 0.868 | 0.000 | 0.024 | - | 20341060 | 0.000 | 0.813 | - | 602360 |
|  | Short arm octopus | Raw | None, Boiling, Blanching, and Stir-frying | 0.000 | 0.868 | 0.000 | 0.003 | - | 154482614 | 0.000 | 0.454 | - | 1078573 |
| Crustacea | Fleshy prawn | Raw | None, Boiling, Grilling, Pan-frying, Airfrying, Parboiling, and Stir-frying | 0.000 | 0.868 | 0.000 | 0.015 | - | 32239330 | 0.000 | 0.753 | - | 650477 |
|  | Shiba shrimp | Raw | None, Boiling, Grilling, Pan-frying, Airfrying, Parboiling, and Stir-frying | 0.000 | 0.868 | 0.000 | 0.013 | - | 36608542 | 0.000 | 0.198 | - | 2476592 |
|  | Whiteleg Shrimp | Boiled-frozen | None, Boiling, Grilling, Pan-frying, Airfrying, Stir-frying, and Parboiling | 0.000 | 0.868 | 0.000 | 0.000 | - | 5947580645 | 0.000 | 0.065 | - | 7522632 |
|  | Shrimp | Salted | None, Boiling, Stir-frying, and Steaming | 0.000 | 0.868 | 0.000 | 0.004 | - | 110103972 | 0.000 | 0.036 | - | 13629517 |
|  | Shrimp | Boiled-dried | None, Boiling, and Stir-frying | 1.107 | 1.701 | 0.004 | 0.006 | 135341634 | 88055967 | 0.088 | 0.135 | 5588920 | 3636263 |
|  | Blue crab (female) | Raw | None, Boiling, and Steaming | 0.000 | 0.868 | 0.000 | 0.020 | - | 24405695 | 0.000 | 0.502 | - | 976646 |
|  | Blue crab (male) | Raw | None, Boiling, and Steaming | 0.000 | 0.868 | 0.000 | 0.002 | - | 212143004 | 0.000 | 2.295 | - | 213477 |
|  | Snow crab | Raw | None, Boiling, and Steaming | 0.000 | 0.868 | 0.000 | 0.004 | - | 132325990 | 0.000 | 1.877 | - | 261066 |
| Sea algae | Laver | Dried | None, Grilling, and Airfrying | 0.178 | 0.923 | 0.001 | 0.005 | 472485809 | 91214414 | 0.008 | 0.039 | 64476837 | 12447394 |
|  | Laver | Roasted | None | 0.144 | 0.921 | 0.000 | 0.003 | 1199198702 | 187039762 | 0.005 | 0.033 | 93963721 | 14655580 |
|  | Laver | Roasted-seasoned | None | 0.591 | 1.143 | 0.001 | 0.001 | 678916031 | 351162383 | 0.040 | 0.078 | 12114187 | 6265939 |
|  | Laver | Fried-seasoned | None | 0.166 | 0.943 | 0.002 | 0.012 | 229848570 | 40553829 | 0.018 | 0.101 | 27537512 | 4858640 |
|  | Sea lettuce | Raw | None and Stir-frying | 0.033 | 0.853 | 0.000 | 0.004 | 3313203384 | 127766754 | 0.014 | 0.355 | 35767184 | 1379287 |
|  | Sea lettuce | Dried | None and Stir-frying | 0.396 | 1.009 | 0.000 | 0.001 | 1220658498 | 479107707 | 0.032 | 0.080 | 15527958 | 6094714 |
|  | Kelp | Raw | None | 0.000 | 0.868 | 0.000 | 0.004 | - | 139241018 | 0.000 | 0.596 | - | 821915 |
|  | Kelp | Dried | None, Boiling, and Airfrying | 0.000 | 0.868 | 0.000 | 0.011 | - | 46073387 | 0.000 | 0.171 | - | 2859856 |
|  | Kelp | Dried | None | 0.192 | 0.873 | 0.000 | 0.000 | 7592553003 | 1671921819 | 0.193 | 0.875 | 2543780 | 560154 |
|  | Kelp stock | Steeped | None and Boiling | 0.000 | 0.868 | 0.000 | 0.275 | - | 1783612 | 0.000 | 1.876 | - | 261165 |
|  | Seaweed fulvescens | Raw | None, Boiling, and Pan-frying | 0.528 | 1.279 | 0.000 | 0.001 | 2189490771 | 904031237 | 0.235 | 0.570 | 2081914 | 859613 |
|  | Sea mustard | Raw | None and Boiling | 0.000 | 0.868 | 0.000 | 0.002 | - | 294486752 | 0.000 | 0.050 | - | 9870918 |
|  | Sea mustard | Dried | None, Soaking, and Boiling after stir-frying | 0.000 | 0.868 | 0.000 | 0.010 | - | 47723817 | 0.000 | 0.084 | - | 5804034 |
|  | Sea mustard stem | Raw | None and Stir-frying | 0.000 | 0.868 | 0.000 | 0.001 | - | 845341411 | 0.000 | 0.045 | - | 10871316 |
|  | Sea mustard | Salted | None | 0.000 | 0.868 | 0.000 | 0.000 | - | 1616818039 | 0.000 | 0.062 | - | 7875355 |
|  | Seaweed fusiforme | Raw | None and Blanching | 0.000 | 0.868 | 0.000 | 0.004 | - | 125542599 | 0.000 | 0.564 | - | 869123 |
| Echinodermata | Sea urchin roe | Raw | None | 0.124 | 0.872 | 0.000 | 0.001 | 5905640988 | 839398732 | 0.012 | 0.083 | 41419180 | 5887118 |
|  | Sea cucumber | Dried | None, Soaking, Boiling, and Stir-frying | 175.746 | 175.75 | 0.269 | 0.269 | 1821692 | 1821692 | 79.015 | 79.015 | 6201 | 6201 |
| Tunicata | Sea squirt | Raw | None | 4.101 | 4.405 | 0.013 | 0.014 | 37758654 | 35152821 | 3.626 | 3.895 | 135127 | 125802 |
|  | Warty sea squirt | Raw | None and Boiling | 0.202 | 0.960 | 0.001 | 0.003 | 720285891 | 151799775 | 0.042 | 0.201 | 11574866 | 2439395 |
| Cnidaria | Jellyfish | Raw | None and Blanching | 0.428 | 1.103 | 0.001 | 0.002 | 774970282 | 300841368 | 0.171 | 0.442 | 2857744 | 1109368 |

**Supplementary Table 2. Toxic equivalent quantity (TEQ), daily exposure, and margin of exposure (MOE) of 109 marine products samples under lower bound (LB) and upper bound (UB) by total consumer population and its different age groups**

|  |  |  |  |  |  | Total population | | | | Consumption group | | | |
| --- | --- | --- | --- | --- | --- | --- | --- | --- | --- | --- | --- | --- | --- |
|  |  |  |  | TEQ_B[a]p_ (μg/kg) | | Dietary exposure  (ng/kg/d) | | MOE | | Dietary exposure  (ng/kg/d) | | MOE | |
| Category | Composite sample | State of composite sample | Cooking method | LB | UB | LB | UB | LB | UB | LB | UB | LB | UB |
| Fish | Mackerel | Raw | None, Boiling, Grilling, and Pan-frying | 1.632 | 2.375 | 0.057 | 0.083 | 8606282 | 5913874 | 1.246 | 1.813 | 393391 | 270322 |
|  | Mackerel | Salted | None, Grilling, and Pan-frying | 4.699 | 5.359 | 0.177 | 0.201 | 2774760 | 2432867 | 4.924 | 5.616 | 99504 | 87244 |
|  | Japanese spanish mackerel | Raw | None, Boiling, and Grilling | 0.127 | 1.079 | 0.002 | 0.013 | 322454826 | 37816703 | 0.157 | 1.341 | 3115153 | 365337 |
|  | Atka mackerel | Raw | None, Boiling, Grilling, and Airfrying | 0.223 | 1.185 | 0.001 | 0.007 | 397759193 | 74866510 | 0.236 | 1.254 | 2075661 | 390682 |
|  | Horse mackerel | Raw | None and Boiling | 0.000 | 1.981 | 0 | 0 | - | - | 0 | 0 | - | - |
|  | Brown sole fish | Raw | None, Boiling, Grilling, and Airfrying | 0.000 | 0.990 | 0 | 0.010 | - | 49681980 | 0 | 0.856 | - | 572594 |
|  | Olive flounder | Raw | None and Boiling | 0.000 | 0.990 | 0 | 0.023 | - | 21233932 | 0 | 1.131 | - | 433060 |
|  | Finespotted flounder | Raw | None, Boiling, and Grilling | 0.267 | 1.219 | 0.000 | 0.002 | 988516606 | 216357868 | 0.397 | 1.814 | 1234341 | 270162 |
|  | Summer flounder | Raw | None, Boiling, and Grilling | 0.464 | 1.416 | 0.001 | 0.004 | 351172260 | 114969462 | 0.358 | 1.092 | 1370617 | 448723 |
|  | Alaska pollock | Raw | None and Boiling | 0.000 | 0.990 | 0 | 0.005 | - | 93641845 | 0 | 1.012 | - | 484418 |
|  | Alaska pollock | Frozen | None, Pan-frying, and Airfrying | 0.000 | 0.990 | 0 | 0.024 | - | 20283644 | 0 | 0.798 | - | 613743 |
|  | Alaska pollock | Semi-dried | None, Boiling, and Airfrying | 0.000 | 0.990 | 0 | 0.012 | - | 40919898 | 0 | 0.984 | - | 498219 |
|  | Alaska pollock | Dried | None, Boiling, and Grilling | 3.757 | 3.862 | 0.056 | 0.057 | 8791760 | 8554182 | 1.350 | 1.388 | 362919 | 353112 |
|  | Alaska pollock roe | Raw | None and Boiling | 0.000 | 0.990 | 0 | 0.001 | - | 541524364 | 0 | 0.534 | - | 917223 |
|  | Alaska pollock roe | Salted | None | 0.000 | 0.990 | 0 | 0.001 | - | 383046761 | 0 | 0.200 | - | 2454005 |
|  | Alaska pollack tripe | Salted | None | 0.010 | 0.992 | 0.000 | 0.001 | 66890689213 | 700726352 | 0.001 | 0.111 | 421413686 | 4414601 |
|  | Pacific cod | Raw | None, Boiling, and Airfrying | 0.000 | 0.990 | 0 | 0.008 | - | 61012047 | 0 | 0.810 | - | 604897 |
|  | Pacific cod | Dried | None | 0.043 | 1.015 | 0.000 | 0.003 | 4063126873 | 172042041 | 0.038 | 0.906 | 12774134 | 540886 |
|  | Pacific cod roe | Raw | None and Boiling | 0.000 | 0.990 | 0 | 0.001 | - | 641498092 | 0 | 0.271 | - | 1810734 |
|  | Pacific cod roe and milt | Raw | None | 0.000 | 0.990 | 0 | 0.000 | - | 1053724303 | 0 | 0.307 | - | 1598321 |
|  | Brown croaker | Raw | None, Boiling, and Grilling | 0.114 | 1.066 | 0.000 | 0.002 | 2605101350 | 278044180 | 0.109 | 1.017 | 4515923 | 481987 |
|  | Yellow croaker | Raw | None, Boiling, Grilling, Pan-frying, and Steaming | 0.291 | 1.259 | 0.006 | 0.024 | 87755667 | 20287220 | 0.176 | 0.760 | 2789204 | 644804 |
|  | Yellow croaker | Salt-dried | None, Boiling, Steaming, and Grilling | 0.168 | 1.130 | 0.002 | 0.013 | 263198307 | 39176681 | 0.161 | 1.085 | 3034065 | 451616 |
|  | Silver pomfret | Raw | None, Boiling, Airfrying, and Grilling | 0.000 | 0.990 | 0 | 0.001 | - | 669453284 | 0 | 0.516 | - | 949507 |
|  | Japanese icefish | Dried | None, Grilling, and Stir-frying | 0.014 | 0.996 | 0.000 | 0.004 | 9481283471 | 130931248 | 0.004 | 0.272 | 130446300 | 1801391 |
|  | Anchovy | Raw | None and Boiling | 0.000 | 0.990 | 0 | 0.003 | - | 159672665 | 0 | 0.564 | - | 869126 |
|  | Anchovy | Salted | None | 0.000 | 0.990 | 0 | 0.002 | - | 281196177 | 0 | 0.067 | - | 7306443 |
|  | Anchovy  (Large size) | Dried | None and Steeping | 0.000 | 0.990 | 0 | 0.010 | - | 48720019 | 0 | 0.042 | - | 11705951 |
|  | Anchovy  (Medium size) | Dried | None and Stir-frying | 0.129 | 1.012 | 0.001 | 0.008 | 481042596 | 61436981 | 0.010 | 0.080 | 48039569 | 6135436 |
|  | Anchovy  (Small size) | Dried | None and Stir-frying | 0.000 | 0.990 | 0 | 0.010 | - | 51108673 | 0 | 0.082 | - | 6009677 |
|  | Tuna | Raw | None | 0.000 | 0.990 | 0 | 0.015 | - | 33507247 | 0 | 0.645 | - | 760168 |
|  | Tuna | Canned | None, Boiling, and Stir-frying | 0.000 | 0.990 | 0 | 0.005 | - | 94766764 | 0 | 0.743 | - | 659322 |
|  | Skipjack tuna | Canned | None, Boiling, and Stir-frying | 0.005 | 0.991 | 0.000 | 0.006 | 17303019737 | 82648156 | 0.002 | 0.447 | 229638690 | 1096873 |
|  | Katsuobushi | Smoked | None and Steeping | 16.231 | 16.726 | 0.001 | 0.001 | 342539612 | 332397347 | 0.096 | 0.099 | 5085451 | 4934876 |
|  | Katsuobushi stock | Smoked and steeped | None | 0.000 | 0.639 | 0 | 0.004 | - | 126887628 | 0 | 0.449 | - | 1092456 |
|  | Cutlassfish | Raw | None, Boiling, and Pan-frying | 0.000 | 0.990 | 0 | 0.017 | - | 28368319 | 0 | 0.725 | - | 675841 |
|  | Pacific saury | Raw | None, Boiling, and Pan-frying | 0.000 | 0.990 | 0 | 0.010 | - | 49221186 | 0 | 0.497 | - | 986261 |
|  | Pacific saury | Canned | None and Boiling | 0.000 | 0.990 | 0 | 0.005 | - | 107586300 | 0 | 0.300 | - | 1631411 |
|  | Sailfin sandfish | Raw | None and Boiling | 0.000 | 0.990 | 0 | 0.003 | - | 150067677 | 0 | 0.961 | - | 509994 |
|  | Amur catfish | Raw | None and Boiling | 0.000 | 0.990 | 0 | 0.004 | - | 118075094 | 0 | 1.550 | - | 316044 |
|  | Pond loach | Raw | None and Boiling | 0.000 | 0.990 | 0 | 0.015 | - | 32565171 | 0 | 1.420 | - | 345027 |
|  | Japanese amberjack | Raw | None, Boiling, Grilling, and Airfrying | 0.000 | 0.990 | 0 | 0.001 | - | 969706418 | 0 | 1.019 | - | 480941 |
|  | Pufferfish | Raw | None, Boiling, and Airfrying | 0.000 | 0.990 | 0 | 0.004 | - | 118122878 | 0 | 1.509 | - | 324811 |
|  | Dark banded rockfish | Raw | None, Boiling, and Grilling | 0.000 | 0.990 | 0 | 0.015 | - | 33240136 | 0 | 1.117 | - | 438494 |
|  | Flathead mullet | Raw | None and Boiling | 0.000 | 0.990 | 0 | 0.001 | - | 333198210 | 0 | 1.227 | - | 399286 |
|  | Blackmouth angler | Raw | None and Boiling | 0.000 | 0.990 | 0 | 0.011 | - | 43905179 | 0 | 1.782 | - | 275012 |
|  | Chum salmon | Raw | None, Airfrying, and Pan-frying | 0.000 | 0.990 | 0 | 0.007 | - | 71242771 | 0 | 0.641 | - | 764623 |
|  | Freshwater eel | Raw | None, Boiling, and Grilling | 0.953 | 1.614 | 0.024 | 0.040 | 20774964 | 12271063 | 1.322 | 2.239 | 370517 | 218852 |
|  | Sea eel | Raw | None and Boiling | 0.000 | 0.990 | 0 | 0.005 | - | 98177475 | 0 | 1.043 | - | 469988 |
|  | Gizzard shad | Raw | None, Boiling, and Grilling | 0.008 | 0.996 | 0.000 | 0.007 | 7705023192 | 65744810 | 0.013 | 1.572 | 36522502 | 311636 |
|  | Filefish | Dried | None, Boiling, and Grilling | 1.227 | 2.103 | 0.006 | 0.010 | 81984223 | 47839053 | 0.462 | 0.791 | 1061406 | 619347 |
|  | Ray | Raw | None and Boiling | 0.000 | 0.990 | 0 | 0.001 | - | 555965013 | 0 | 0.703 | - | 697130 |
|  | Skate | Fermented | None, Steaming, and Boiling | 0.000 | 0.990 | 0 | 0.013 | - | 39084311 | 0 | 1.177 | - | 416254 |
|  | Flying fish roe | Raw | None | 0.000 | 0.990 | 0 | 0.000 | - | 1031383858 | 0 | 0.164 | - | 2981681 |
|  | Fish sauce | Salted | None, Boiling, and Stir-frying | 0.000 | 0.639 | 0 | 0.002 | - | 266172752 | 0 | 0.013 | - | 38839146 |
| Shellfish | Clam | Raw | None, Boiling, and Steaming | 0.064 | 1.035 | 0.000 | 0.003 | 2953747792 | 181765097 | 0.014 | 0.225 | 35399040 | 2178355 |
|  | Manila clam | Raw | None and Boiling | 0.099 | 1.036 | 0.002 | 0.023 | 225965484 | 21554610 | 0.019 | 0.195 | 26383165 | 2516662 |
|  | Manila clam | Salted | None | 0.268 | 1.143 | 0.000 | 0.001 | 3024517768 | 708647063 | 0.033 | 0.142 | 14717072 | 3448222 |
|  | Marsh clam | Raw | None and Boiling | 0.481 | 1.381 | 0.000 | 0.001 | 2347786226 | 817794771 | 0.066 | 0.190 | 7412281 | 2581889 |
|  | Scallop | Raw | None, Boiling, and Grilling | 0.184 | 1.072 | 0.001 | 0.007 | 406178815 | 69626173 | 0.283 | 1.649 | 1733110 | 297085 |
|  | Oyster | Raw | None, Boiling, and Pan-frying | 0.161 | 1.131 | 0.002 | 0.016 | 209414567 | 29800169 | 0.085 | 0.596 | 5774201 | 821682 |
|  | Oyster | Salted | None | 0.299 | 1.188 | 0.000 | 0.001 | 1843097298 | 463632454 | 0.126 | 0.502 | 3882015 | 976524 |
|  | Blood cockle | Raw | None, Boiling, and Parboiling | 0.032 | 0.978 | 0.000 | 0.008 | 1899767713 | 62682156 | 0.037 | 1.116 | 13308622 | 439113 |
|  | Pen shell | Raw | None, Boiling, and Grilling | 0.045 | 1.027 | 0.000 | 0.002 | 5239365110 | 231917937 | 0.034 | 0.758 | 14598023 | 646174 |
|  | Red shell | Raw | None and Boiling | 0.226 | 1.111 | 0.000 | 0.001 | 1976560723 | 402119766 | 0.107 | 0.525 | 4591163 | 934045 |
|  | Mussel | Raw | None, Boiling, Pan-frying, and Stir-frying | 0.067 | 1.039 | 0.001 | 0.011 | 666363851 | 42873445 | 0.026 | 0.399 | 19091505 | 1228336 |
|  | Mussel | Boiled-dried | None, Stir-frying after soaking, Pan-frying after soaking, and Boiling | 0.309 | 1.267 | 0.001 | 0.002 | 935370985 | 228008870 | 0.040 | 0.163 | 12337939 | 3007534 |
|  | Whelk | Raw | None, Boiling, and Parboiling | 0.002 | 0.990 | 0.000 | 0.001 | 178816892634 | 449986909 | 0.002 | 0.771 | 252438023 | 635252 |
|  | Whelk | Canned | None | 0.000 | 0.990 | 0 | 0.001 | - | 449740573 | 0 | 0.772 | - | 634904 |
|  | Abalone | Raw | None, Boiling, Pan-frying, and Steaming | 0.000 | 0.990 | 0 | 0.005 | - | 97131991 | 0 | 0.244 | - | 2008198 |
|  | Freshwater snail | Raw | None, Boiling, and Parboiling | 0.000 | 0.990 | 0 | 0.001 | - | 444941512 | 0 | 0.099 | - | 4931508 |
|  | Conch | Raw | None, Parboiling, Boiling after parboiling, and Stir-frying after parboiling | 0.000 | 0.990 | 0 | 0.001 | - | 590853506 | 0 | 0.169 | - | 2894175 |
| Cephalopoda | Squid | Raw | None, Boiling, Pan-frying, Airfrying, Stir-frying, and Parboiling | 0.013 | 0.991 | 0.001 | 0.068 | 536758700 | 7208297 | 0.007 | 0.497 | 73475574 | 986726 |
|  | Squid | Dried | None and Grilling | 0.197 | 1.121 | 0.004 | 0.021 | 132195041 | 23249923 | 0.111 | 0.628 | 4434091 | 779850 |
|  | Squid | Salted | None | 0.009 | 0.991 | 0.000 | 0.003 | 19689776193 | 188722260 | 0.001 | 0.105 | 488345562 | 4680687 |
|  | Cuttlefish | Raw | None, Stir-frying, Pan-frying, and Blanching | 0.006 | 0.993 | 0.000 | 0.001 | 73072687160 | 439587860 | 0.002 | 0.390 | 208822851 | 1256228 |
|  | Octopus | Raw | None, Boiling, and Parboiling | 0.000 | 0.990 | 0 | 0.006 | - | 81168418 | 0 | 0.349 | - | 1404925 |
|  | Octopus | Dried | None and Grilling | 0.328 | 1.217 | 0.000 | 0.001 | 1576319654 | 424944000 | 0.154 | 0.573 | 3172179 | 855155 |
|  | Small octopus | Raw | None, Boiling, and Stir-frying | 0.000 | 0.990 | 0 | 0.027 | - | 17825921 | 0 | 0.860 | - | 569662 |
|  | Short arm octopus | Raw | None, Boiling, Blanching, and Stir-frying | 0.000 | 0.990 | 0 | 0.004 | - | 135381091 | 0 | 0.485 | - | 1009612 |
| Crustacea | Fleshy prawn | Raw | None, Boiling, Grilling, Pan-frying, Airfrying, Parboiling, and Stir-frying | 0.000 | 0.990 | 0 | 0.017 | - | 28252989 | 0 | 0.855 | - | 573235 |
|  | Shiba shrimp | Raw | None, Boiling, Grilling, Pan-frying, Airfrying, Parboiling, and Stir-frying | 0.000 | 0.990 | 0 | 0.015 | - | 32081956 | 0 | 0.220 | - | 2226648 |
|  | Whiteleg Shrimp | Boiled-frozen | None, Boiling, Grilling, Pan-frying, Airfrying, Stir-frying, and Parboiling | 0.000 | 0.990 | 0 | 0.000 | - | 5212171999 | 0 | 0.076 | - | 6482968 |
|  | Shrimp | Salted | None, Boiling, Stir-frying, and Steaming | 0.000 | 0.990 | 0 | 0.005 | - | 96489796 | 0 | 0.040 | - | 12104181 |
|  | Shrimp | Boiled-dried | None, Boiling, and Stir-frying | 0.059 | 1.032 | 0.000 | 0.003 | 2546136377 | 145082677 | 0.005 | 0.082 | 105409773 | 6006407 |
|  | Blue crab (female) | Raw | None, Boiling, and Steaming | 0.000 | 0.990 | 0 | 0.023 | - | 21387970 | 0 | 0.545 | - | 899405 |
|  | Blue crab (male) | Raw | None, Boiling, and Steaming | 0.000 | 0.990 | 0 | 0.003 | - | 185911867 | 0 | 2.923 | - | 167644 |
|  | Snow crab | Raw | None, Boiling, and Steaming | 0.000 | 0.990 | 0 | 0.004 | - | 115964097 | 0 | 2.224 | - | 220293 |
| Sea algae | Laver | Dried | None, Grilling, and Airfrying | 0.018 | 0.996 | 0.000 | 0.006 | 4724858086 | 84550838 | 0.001 | 0.042 | 649358243 | 11620197 |
|  | Laver | Roasted | None | 0.014 | 0.996 | 0.000 | 0.003 | 11991987020 | 172924488 | 0.001 | 0.036 | 954448444 | 13763149 |
|  | Laver | Roasted-seasoned | None | 0.059 | 1.018 | 0.001 | 0.013 | 646928986 | 37584004 | 0.004 | 0.072 | 117815597 | 6844618 |
|  | Laver | Fried-seasoned | None | 0.017 | 0.998 | 0.000 | 0.001 | 24121330493 | 402325924 | 0.002 | 0.124 | 237522708 | 3961703 |
|  | Sea lettuce | Raw | None and Stir-frying | 0.003 | 0.989 | 0.000 | 0.004 | 33132033838 | 110187558 | 0.001 | 0.399 | 368826353 | 1226610 |
|  | Sea lettuce | Dried | None and Stir-frying | 0.070 | 1.003 | 0.000 | 0.001 | 6932795748 | 481901274 | 0.005 | 0.072 | 98176989 | 6824320 |
|  | Kelp | Raw | None | 0.000 | 0.990 | 0 | 0.004 | - | 122024094 | 0 | 0.681 | - | 719188 |
|  | Kelp | Dried | None, Boiling, and Airfrying | 0.000 | 0.990 | 0 | 0.012 | - | 40376488 | 0 | 0.211 | - | 2323581 |
|  | Kelp | Dried | None | 0.019 | 0.991 | 0.000 | 0.000 | 75925530026 | 1473360222 | 0.020 | 1.031 | 24497892 | 475390 |
|  | Kelp stock | Steeped | None and Boiling | 0.000 | 0.639 | 0 | 0.202 | - | 2421748 | 0 | 1.328 | - | 368849 |
|  | Seaweed fulvescens | Raw | None, Boiling, and Pan-frying | 0.005 | 0.995 | 0.000 | 0.000 | 218949077145 | 1162699981 | 0.003 | 0.482 | 191394951 | 1016377 |
|  | Sea mustard | Raw | None and Boiling | 0.000 | 0.990 | 0 | 0.002 | - | 258073945 | 0 | 0.054 | - | 9134231 |
|  | Sea mustard | Dried | None, Soaking, and Boiling after stir-frying | 0.000 | 0.990 | 0 | 0.012 | - | 41822845 | 0 | 0.101 | - | 4859643 |
|  | Sea mustard stem | Raw | None and Stir-frying | 0.000 | 0.990 | 0 | 0.001 | - | 740816325 | 0 | 0.051 | - | 9575539 |
|  | Sea mustard | Salted | None | 0.000 | 0.990 | 0 | 0.000 | - | 1416901126 | 0 | 0.077 | - | 6396244 |
|  | Seaweed fusiforme | Raw | None and Blanching | 0.000 | 0.990 | 0 | 0.004 | - | 122279695 | 0 | 0.611 | - | 802451 |
| Echinodermata | Sea urchin roe | Raw | None | 0.012 | 0.991 | 0.000 | 0.001 | 59056409878 | 738647599 | 0.001 | 0.083 | 473091272 | 5917186 |
|  | Sea cucumber | Dried | None, Soaking, Boiling, and Stir-frying | 31.677 | 31.677 | 0.048 | 0.048 | 10106947 | 10106947 | 12.678 | 12.678 | 38651 | 38651 |
| Tunicata | Sea squirt | Raw | None | 0.527 | 1.415 | 0.002 | 0.004 | 293745121 | 109428992 | 0.442 | 1.185 | 1109849 | 413452 |
|  | Warty sea squirt | Raw | None and Boiling | 0.020 | 1.000 | 0.000 | 0.003 | 7202858914 | 145831432 | 0.004 | 0.212 | 114198808 | 2312106 |
| Cnidaria | Jellyfish | Raw | None and Blanching | 0.111 | 1.051 | 0.000 | 0.002 | 2990218999 | 315909864 | 0.042 | 0.399 | 11630250 | 1228710 |

**Supplementary Table 2. Accuracy and precision of PAH8 using two different extraction methods**

| Compounds | Nominal concentration (µg/kg) | Alkali digestion method | | | | Ultrasonication method | | | |
| --- | --- | --- | --- | --- | --- | --- | --- | --- | --- |
|  |  | Intra-day ^a)^ (n=5) | | Inter-day ^b)^ (n=3) | | Intra-day (n=5) | | Inter-day (n=3) | |
|  |  | Accuracy | Precision | Accuracy | Precision | Accuracy | Precision | Accuracy | Precision |
|  |  | (%) | (% RSD ^c)^) | (%) | (% RSD) | (%) | (% RSD) | (%) | (% RSD) |
| B[a]A | 5 | 97.35-100.00 | 1.92-3.42 | 99.74 | 1.09 | 98.43-101.39 | 0.53-2.27 | 100.70 | 0.84 |
|  | 10 | 98.80-101.02 | 1.07-3.41 | 99.79 | 0.62 | 99.54-103.56 | 1.87-2.17 | 100.45 | 0.80 |
|  | 20 | 99.98-106.03 | 1.87-4.08 | 102.13 | 2.28 | 102.18-106.83 | 1.57-2.43 | 102.11 | 1.50 |
| Chry | 5 | 98.30-100.27 | 0.76-0.99 | 99.85 | 0.68 | 98.38-100.72 | 1.05-2.26 | 99.77 | 0.53 |
|  | 10 | 96.75-99.73 | 1.72-3.13 | 99.52 | 0.81 | 98.29-100.83 | 0.82-2.21 | 99.93 | 0.26 |
|  | 20 | 99.50-106.62 | 0.93-7.42 | 100.01 | 0.24 | 102.41-103.05 | 0.80-3.71 | 100.77 | 3.32 |
| B[b]F | 5 | 97.92-98.47 | 1.20-2.59 | 99.93 | 0.47 | 96.82-98.15 | 0.55-1.20 | 98.60 | 0.66 |
|  | 10 | 97.23-97.76 | 2.66-4.86 | 99.58 | 0.42 | 97.78-98.06 | 0.60-0.76 | 98.65 | 0.10 |
|  | 20 | 98.24-105.74 | 1.12-8.35 | 100.15 | 0.08 | 98.35-98.92 | 0.39-0.57 | 99.20 | 0.31 |
| B[k]F | 5 | 99.07-100.30 | 0.87-2.49 | 99.99 | 0.07 | 96.29-100.39 | 1.32-4.22 | 98.82 | 0.60 |
|  | 10 | 98.49-99.64 | 2.32-3.22 | 100.24 | 0.13 | 89.93-96.94 | 2.83-6.72 | 97.81 | 2.43 |
|  | 20 | 99.95-101.91 | 0.24-1.25 | 100.23 | 0.38 | 96.26-97.18 | 1.12-2.60 | 98.74 | 0.89 |
| B[a]P | 5 | 98.80-100.52 | 1.03-1.87 | 99.96 | 0.25 | 98.79-101.00 | 1.20-3.76 | 100.60 | 0.68 |
|  | 10 | 98.31-100.29 | 1.31-3.92 | 100.48 | 0.91 | 96.86-98.77 | 0.75-2.95 | 99.60 | 0.64 |
|  | 20 | 99.98-111.30 | 0.96-8.75 | 100.41 | 0.36 | 97.84-99.72 | 0.65-1.27 | 99.86 | 0.48 |
| I[c,d]P | 5 | 98.92-102.63 | 1.13-4.87 | 99.76 | 0.75 | 91.83-93.76 | 0.64-1.77 | 94.44 | 1.71 |
|  | 10 | 98.25-101.87 | 1.94-4.27 | 100.27 | 0.38 | 94.07-96.76 | 2.29-2.85 | 98.19 | 1.47 |
|  | 20 | 101.47-105.78 | 1.38-3.31 | 101.17 | 1.55 | 94.20-95.55 | 1.97-2.44 | 97.54 | 1.69 |
| D[a,h]A | 5 | 99.85-105.53 | 0.78-7.00 | 101.37 | 0.71 | 94.62-96.16 | 1.53-7.36 | 98.88 | 1.65 |
|  | 10 | 100.32-107.50 | 0.51-5.29 | 100.92 | 0.63 | 98.53-104.07 | 1.98-3.86 | 99.82 | 0.89 |
|  | 20 | 102.97-111.82 | 2.88-6.44 | 103.99 | 3.43 | 99.54-106.69 | 1.04-7.22 | 100.47 | 1.12 |
| B[g,h,i]P | 5 | 99.23-105.51 | 1.22-7.10 | 101.03 | 1.41 | 92.71-93.11 | 0.61-1.36 | 94.01 | 0.69 |
|  | 10 | 104.06-109.97 | 2.77-4.49 | 102.13 | 1.93 | 95.60-96.21 | 1.15-2.50 | 97.70 | 1.10 |
|  | 20 | 100.83-108.58 | 1.67-5.75 | 101.38 | 0.87 | 98.29-100.09 | 1.18-1.85 | 99.68 | 1.15 |

^a)^ ranged from mean of 5 determinations performed daily for 3 days.

^b)^ mean of 3 determinations.

^c)^ relative standard deviation: 100 x standard deviation/mean.
